# Copy number variants (CNVs): a powerful tool for iPSC-based modelling of ASD

**DOI:** 10.1186/s13229-020-00343-4

**Published:** 2020-06-01

**Authors:** Danijela Drakulic, Srdjan Djurovic, Yasir Ahmed Syed, Sebastiano Trattaro, Nicolò Caporale, Anna Falk, Rivka Ofir, Vivi M. Heine, Samuel J. R. A. Chawner, Antonio Rodriguez-Moreno, Marianne B. M. van den Bree, Giuseppe Testa, Spyros Petrakis, Adrian J. Harwood

**Affiliations:** 1 Institute of Molecular Genetics and Genetic Engineering, University of Belgrade, 11042 Belgrade, 152 Serbia; 2 Department of Medical Genetics, Oslo University Hospital, 0424 Oslo, Norway; 3 NORMENT, Department of Clinical Science, University of Bergen, 5007 Bergen, Norway; 4 Neuroscience & Mental Health Research Institute, Cardiff University, Cardiff, CF24 4HQ UK; 5 Laboratory of Stem Cell Epigenetics, IEO, European Institute of Oncology, IRCCS, 20146 Milan, Italy; 6 Department of Oncology and Hemato-oncology, University of Milan, 20122 Milan, Italy; 7 Department of Neuroscience, Karolinska Institutet, 17177 Stockholm, Sweden; 8 BGU-iPSC Core Facility, The Regenerative Medicine & Stem Cell (RMSC) Research Center, Ben Gurion University of the Negev, 84105 Beer-Sheva, Israel; 9 Complex Trait Genetics, Center for Neurogenomics and Cognitive Research, Amsterdam Neuroscience, Vrije Universiteit Amsterdam, Amsterdam, The Netherlands; 10 Child and Youth Psychiatry, Emma Children’s Hospital, Amsterdam UMC, Amsterdam Neuroscience, Vrije Universiteit Amsterdam, 1081 Amsterdam, The Netherlands; 11 MRC Centre for Neuropsychiatric Genetics and Genomics, Cardiff University, Cardiff, CF24 4HQ UK; 12 Department of Physiology, Anatomy and Cell Biology, University Pablo de Olavide, Ctra. de Utrera, Km 1, 41013 Seville, Spain; 13 Human Technopole, Via Cristina Belgioioso 171, 20157 Milan, Italy; 14 Institute of Applied Biosciences/Centre for Research and Technology Hellas, 57001 Thessaloniki, Greece

**Keywords:** Human iPSCs, Copy number variants (CNVs), Neurodevelopmental disorders (NDD), Autism spectrum disorders (ASD)

## Abstract

Patients diagnosed with chromosome microdeletions or duplications, known as copy number variants (CNVs), present a unique opportunity to investigate the relationship between patient genotype and cell phenotype. CNVs have high genetic penetrance and give a good correlation between gene locus and patient clinical phenotype. This is especially effective for the study of patients with neurodevelopmental disorders (NDD), including those falling within the autism spectrum disorders (ASD). A key question is whether this correlation between genetics and clinical presentation at the level of the patient can be translated to the cell phenotypes arising from the neurodevelopment of patient induced pluripotent stem cells (iPSCs).

Here, we examine how iPSCs derived from ASD patients with an associated CNV inform our understanding of the genetic and biological mechanisms underlying the aetiology of ASD. We consider selection of genetically characterised patient iPSCs; use of appropriate control lines; aspects of human neurocellular biology that can capture in vitro the patient clinical phenotype; and current limitations of patient iPSC-based studies. Finally, we consider how future research may be enhanced to maximise the utility of CNV patients for research of pathological mechanisms or therapeutic targets.

## Background

Autism spectrum disorders (ASD) are a complex and heterogeneous group within the wider spectrum of neurodevelopmental disorders (NDD), which in its entirety also encompass intellectual disability (ID), attention-deficit/hyperactivity disorders (ADHD) and schizophrenia. ASD in particular are characterised by deficits in social interaction, communication difficulty and the presence of restricted, repetitive and stereotyped patterns of behaviour [1]. However, individual autistic patients often have features of other NDD, as well as comorbidities, such as epilepsy and anxiety [2]. As a consequence, their study therefore can provide insights across a range of common psychiatric disorders.

As seen with other NDD, the underlying biological origins of ASD begin during brain development and neural maturation; however, the clinical symptoms only emerge progressively during infancy, and as a consequence it is often difficult to diagnose at early post-natal stages. In addition, the spectrum of symptoms and phenotypes vary considerably from one individual to the next, presenting a challenge to identify those changes that directly arise from early developmental deficits and those that accumulate either indirectly or due to the effects of external factors. These complications hinder identification of the basic pathophysiological mechanisms that lead to ASD and hence hamper development of effective therapies.

Molecular and cellular analysis of human patients is generally prospective with data mostly derived from post-mortem tissue. As mentioned above, such studies are subject to the confounds of secondary effects and record the outcomes of underlying disease mechanism rather than directly probe the causative mechanisms. Animal models can be highly informative for the study of a basic mechanism; however, it is difficult to directly translate between observed patient phenotype and animal models. A particular weakness is the ability to capture the phenotypic variation across the patient population.

Human stem cell models offer an opportunity to directly study the molecular and cellular mechanisms of diseases. Key to this approach is the generation of human-induced pluripotent stem cells (iPSCs) derived from patient cells. These are generated by reprogramming of somatic cells into pluripotent stem cells from which many cell types can be differentiated, including neurons and glial cells. Importantly, they can be easily obtained in the clinic from fibroblasts (skin biopsies), keratinocytes (hair roots) [3], T lymphocytes (peripheral blood) [4, 5] and exfoliated renal epithelial cells from urine samples [6, 7]. Importantly, patient iPSCs enable the in vitro study of different cells types in isolation or co-culture in order to investigate cell function. Uniquely they can track the development profile of patient cell differentiation. More recently the capacity of iPSCs to form 3D organoids has opened up the possibility to investigate the interaction of multiple cell types in a more brain-like microenvironment. Methods for increasing reproducibility of brain organoid differentiation are improving substantially [8, 9] and being exploited to mechanistically dissect the effect of genetic lesions causing ASD and ID [10–12], as well as the role of specific genes and molecular modules key to human-specific neuronal differentiation trajectories and pathophysiology [13].

The major question is how to identify the relevant cellular phenotypes that converge on the common pathophysiological mechanisms underlying patient aetiology. Recent technical advances, particularly the advent of microarray technologies and whole-genome sequencing (WGS), have heralded a new era for detection of genetic risk loci for ASD [14, 15]. Unfortunately, most genetic risk for ASD is due to variations on 100’s of loci dispersed across the genome, each contributing only a small component to the overall level of genetic risk. Over the last decade, an accumulation of genetic evidence has pointed to three broad aspects of neuronal cell biology associated with elevated risk: synapse biology, gene regulation and neuro-inflammatory pathways. Emerging studies indicate that this dysregulated cell physiology contributes to circuit dysfunction, cortical layer malformation and white matter alteration seen within the patient brain. All of these biological processes and functional pathways can be investigated in patient iPSCs, offering capabilities beyond studies of post-mortem tissue or preclinical rodent models.

The dispersed nature of common genetic risk creates difficulties for cell modelling with most patient iPSCs. Although common genetic risk can be calculated as an overall polygenic risk score (PRS), this does not provide a direct concordance between specific changes at a genetic locus and the symptoms associated with ASD. In contrast, single nucleotide variants (SNVs) and copy number variants (CNVs) have much higher genetic penetrance for risk of developing ASD and other NDD [16] and hence changes at single or a few loci may make a major contribution to the clinical phenotype. Both SNVs and CNVs are a lot less common within the general patient population, but at present common clinical availability of array comparative genomic hybridisation (aCGH) technology makes CNVs more likely to be identified by cytogenomic screening. Accordingly, patients harbouring pathogenic CNVs present a powerful opportunity to relate genetic risk to patient clinical presentation. Here, we examine how iPSC-based studies using CNV patients can provide insights into the relationship of risk genetics to biological outcomes and can be utilised for the elucidation of disease mechanism.

## Main text

An estimated 5–10% of all ASD cases carry CNVs [14], compared to 1.4–2.5% of all schizophrenia cases [15, 17]. Often CNVs arise spontaneously or de novo, although they can also be inherited in families, and at least 90 pathogenic CNVs have been reported for ASD [18]. There is however a core of CNVs associated with strong evidence of association with ASD and prevalent amongst people referred for genetic testing (Table 1) [19–22]. It is clear that despite having high genetic penetrance, CNVs present with considerable clinical variation in severity, phenotypic profile and co-morbidity, even between individuals with CNVs at the same chromosomal locus. For example, it is well established that a deletion at the 22q11.2 locus (22q11.2DS) is associated with ASD, but when children were identified first by genetic screening as few as 16% had ASD using stringent assessment criteria, although more than 80% showed diagnostic criteria for at least one psychiatric disorder and approximately 60% exhibited characteristics of ADHD [23]. In adults, 25% of patients with 22q11.2DS develop schizophrenia, 70% of the individuals possessing the same size deletion at the 22q11.2 locus exhibit congenital heart conditions [24–26].

**Table 1:** Frequent CNVs associated with risk for neurodevelopmental disorders (NDDs)

Locus	Syndrome	Rearrangements	Position of critical region	Key genes	% in People with Autism
1q21.1		1q21.1; del and dup	chr1:146,527,987-147,394,444	*HYDIN2*, *PRKAB2*, *CHD1L*, *BCL9*, *GJA5*, *GJA8*, *GPR89B*	*Del 0*.*039%*, *Dup 0*.*157%* (Pinto et al., 2014) [19]
2p16.3		del	chr2:50145643-51259674	*NRXN1*	*Del 0*.*316%* (Pinto et al., 2014) [19]
3q29		del	chr3:195,720,167-197,354,826	*DLG1*	*Del 0*.*005%* (Malhotra et al., 2012) [20]
7q11.23	Williams-Beuren syndrome (WBS)	del and dup	chr7:72,744,915-74,142,892	*CLDN3*, *CLDN4*, *GTF2*, *ELN*, *LIMK1*, *KCTD7*, *CLIP2*, *STX1A*,	*Del* .*024%*, *Dup 0*.*097%* (Pinto et al., 2014) [19]
9q34	Kleefstra syndrome	del	chr9:140,513,444-140,730,578	*EHMT1*	*Del 0*.*049%* (Pinto et al., 2014) [19]
15q11.2		BP1-BP2; del and dup	chr15:22,805,313-23,094,530	*CYFIP1*	*Del 0*.*09%* (Malhotra et al., 2012) [20], *Dup 0*.*94%* (van der Zwaag et al., 2010) [21]
15q11-q13	Prader-Willi and Angleman’s syndromes	BP2-BP3	chr15:29,161,368-32462776	*UBE3A*, *ATP10A*, *GABARB3*, *GABARA5*, *GABARG3*	*Del 0*.*192%* (Depienne et al., 2009) [22], *Dup 0*.*255%* (Pinto et al., 2014) [19]
15q13.3		BP4-BP5; del and dup	chr15:32,017,070-32,453,068	*CHRNA7*	*Del 0*.*157%*, *Dup 0*.*039%* (Pinto et al., 2014) [19]
16p13.11		del and dup	chr16:15,511,655-16,293,689	*NDE1*, *MYH11*	*Del 0*.*137%*, *Dup 0*.*268%* (Pinto et al., 2014) [19]
16p11.2		proximal (593 kb) del and dup	chr16:29,650,840-30,200,773	*KCTD13*, *ALDOA*, *CORO1A*, *MAPK3*, *TAOK2*	*Del 0*.*42%*, *Dup 0*.*39%* (Malhotra et al., 2012) [20]
17q12	Renal cysts and diabetes syndrome (RCAD)	del and dup	chr17:34,815,904-36,217,432	*NF1*	*Del 0*.*039%*, *Dup 0*.*020%* (Pinto et al., 2014) [19]
22q11.2	Deletion known as DiGeorge syndrome, Velocardiofacial syndrome and 22q11.2 deletion syndrome	del and dup	chr22:19,037,332-21,466,726	*TBX1*, *COMT*, *PI4KA*, *SEPT6*	*Del 0*.*059%*, *Dup 0*.*157%* (Pinto et al., 2014) [19]
22q13	Phelan-McDermid syndrome (PMDS)	del	chr22:51113070-51171640	*SHANK3*	*Del 0* .*097%* (Pinto et al., 2014) [19]

These observations define the key questions of both genetics and biology that CNV patient iPSCs can address by the development of cell modelling of ASD and other NDD. In this review, we will discuss how these studies help understand the mechanisms underlying the genotype-to-phenotype relationship for ASD risk; what aspects of ASD can be meaningfully modelled in iPSC-derived neurons, and what limitations these studies possess. Finally, we will consider what methodological approaches are required to advance these studies.

## Genotype-to-phenotype relationship in NDD patients harbouring pathogenic CNVs

The possible reasons for variation in the relationship between genotype and phenotype for different patients are still uncertain; however, they present an important consideration when choosing which patients to select for further study. What additional genetic factors and genomic mechanisms might increase phenotypic variation of patients with apparent similar risk loci?

First, the size, and hence number of genes affected at individual loci can vary considerably, with many of the larger CNVs having a number of different break points. The 22q11.2 locus for example can occur between two of four different breakpoints (A–D) with five different forms of deletion reported in the patient population [24]. In addition, break point regions can have complex local sequence changes, such as short sequence inversions or insertions and deletions (indels) that vary from one individual to the next but are not detected by the commonly used sequencing technologies and arrays [27]. With the advent of a new generation of single-molecule real-time (SMRT) sequence technologies that are capable of very long reads in each run, this level of variation is likely to be resolved in the future. Furthermore, it is also becoming very clear that the 3D chromatin structure, such as chromosome loops (topological associated domains, TADs) and long-range chromatin interactions, also play an important role in gene regulation. CNVs may influence or even disrupt gene regulation beyond the specific sequences contained within them [28]. Again, techniques are becoming available to accurately map these changes. Although these sources of genome variation are still a major challenge for CNV analysis, they also present a considerable opportunity for iPSC-based studies due to their ability to draw together genomics, transcriptomics and quantitative cell phenotyping.

A second major source of genomic variation arises due to patient diagnosis. As in the great majority of published studies patients are initially selected on the basis of their clinical presentation, for example congenital abnormality or developmental delay, which leads to then being referred for genetic testing. As a consequence, there is likely to be acquisition bias and under-representation of those individuals who have little or no pathology associated with their CNV. This bias may select for patients who possess additional background variation in their genome that enhances the effect of a CNV but is not detected by standard clinical screening. Studies on the impact of carrying several “pathogenic” CNVs indicate that children who carried two large CNVs of unknown clinical significance were eight times as likely to have developmental delay than patients with a single CNV [29]. Observations of the 16p12.1 deletion suggest a two-hit model for severity of its associated impact on developmental delay [30]. Possession of common risk variants in an individual background genome may also influence severity and clinical presentation. A study on schizophrenia patients showed higher PRS in individuals with low-penetrant CNVs in comparison to those with high-penetrant CNVs [31] and children with ADHD showed lower PRS when carrying large, rare CNVs in comparison to children with ADHD without such CNVs [32]. These results support the proposition that CNVs exert the same genetic pressure on risk as common variants but in general are more penetrant.

Finally, when using patient iPSCs for modelling it is important to take into account that for some CNVs, ASD is 4-fold less prevalent in females than males, yet there is an excess of deleterious CNVs in the female population, suggesting the existence of a protective effect [33]. It is currently unclear whether this protection extends in cells derived from female patients after reprogramming and/or during in vitro neuro-differentiation but needs to be considered as a possible confound when comparing between cell lines from different patients and non-patient controls.

## Appropriate controls for patient iPSC studies

The genetic observations above underlie the need for careful selection of controls for iPSC studies. In an ideal situation, iPSCs derived from parents and siblings should be used to generate control cells alongside patient cells. In addition, genome editing techniques address the need for controls by standardising genetic backgrounds via generation of isogenic cell lines. Here, CRISPR/Cas9-mediated genome editing can be used to target a single gene within a CNV, so that the edited iPSC lines will have an identical genetic background to the parental line, minimising heterogeneity and phenotypic variability arising due to the genetic differences in the genomic background [34]. Multiple rounds of CRISPR can be used to sequentially disrupt more than one gene to model aspects of larger CNVs, or pairs of gRNA used to generate large genomic deletions and other rearrangements to create cell models with up to 1Mbp deletions [35] or reciprocal CNVs in human iPSCs [36]. In all cases, care should be taken to avoid introduction of off-target mutations leading to the small indels or even CNVs elsewhere in the genome. As a minimum, it is advisable to study multiple, independent engineered cells lines and genotype each using array screening. In future, availability and prevalence of WGS technologies may allow for more in-depth analysis. Finally, ideally engineered cells lines can be “rescued” by further engineering or reversible transgenesis using a *piggyBac* transposon system [37] to the original gene copy number.

## What aspects of ASD can be modelled in iPSCs derived from CNV patients?

Although not without the challenges described above, a high degree of genetic penetrance makes a strong biological case for use of iPSCs from patients harbouring pathogenic CNVs as the basis for creation of disease-relevant cell assays. However, beyond a simple justification of providing access to human cell physiology, patient iPSC studies need to be tailored to align with the underlying biology observed for ASD and other NDD. Table 2 (and Supplementary Tables 2a and 2b) list those iPSC lines and their analysis that have been reported to date.

**Table 2:** iPSCs derived from patients with ASD associated CNVs

CNV	Type and size of CNV	Source	Reprogramming	Number of patients and healthy controls	Differentiation protocol	Neuronal cell types	Associated cellular phenotype	Ref.
1p21.3	Deletion Size of CNV—not available in the paper	Keratinocytes	Patient: CytoTune-iPS Sendai Reprogramming Kit Controls: Constitutive Polycistronic Lentivirus Reprogramming Kit	Patient: 1 Controls: 3	Neurons (cortical neuron differentiation method based on dual SMAD inhibition)	Neural precursors Neural cells	Delay in expression of neuronal markers Dynamic imbalance in GABA/glutamate cell populations over time Enrichment of gene networks identified in autism post-mortem brains	Adhya et al., 2019 [38]
1p33	323 kb deletion/+ (chr1:49894000-50224000del) (primary genetic variant) Other ASD implicated variants detected in patient: - 2q21.1 516 kb duplication/+ - HTR3A p.G148X/+	Fibroblasts	CytoTune-iPS Sendai Reprogramming Kit	Patient: 1 Controls: 11 (total in the study—controls: 11; ASD-affected: 14)	Neurons (NGN2 ectopic expression approach)	Glutamatergic neurons	Reduced weighted mean firing rate	Deneault et al., 2019 [39]
2p16.3	Bi-allelic *NRXN1*-*α* deletion - Paternal deletion: exon 1-5, ~ 0.4 kb - Maternal deletion: exon 1-5, ~ 0.18 kb	Fibroblasts	CytoTune-iPS reprogramming kit	Patient: 1 Controls: 4	Neuroepithelial stem cells Neurons (modified dual SMAD inhibition protocol)	Neuroepithelial stem cells Neurons	Slower proliferation rate Expression of radial glia-like genes and preferentially differentiation to astroglia Depressed calcium signalling capacity, lower levels of neurotransmitter, impairment of maturation	Lam et al., 2019 [40]
2p16.3	Deletion Size of CNV—not available in the paper	Keratinocytes	Patients: CytoTune-iPS Sendai Reprogramming Kit Controls: Constitutive Polycistronic Lentivirus Reprogramming Kit	Patients: 2 Controls: 3	Neurons (cortical neuron differentiation method based on dual SMAD inhibition)	Neural precursors Neural cells	Delay in expression of neuronal markers Dynamic imbalance in GABA/glutamate cell populations over time Enrichment of gene networks identified in autism post-mortem brains	Adhya et al., 2019 [38]
2p16.3	De novo 430 kb deletion/+ (chr2:50567944-51057790del)	Fibroblasts	Retroviruses expressing *OCT4/POU5F1*, *SOX2*, *KLF4* and *MYC* and lentiviral vector containing pluripotency reporter EOS-GFP/PuroR	Patient: 1 Controls: 11 (total in the study—controls: 11; ASD-affected: 14)	Neurons (NGN2 ectopic expression approach)	Glutamatergic neurons	No differences in weighted mean firing rate between patient and pool of all controls	Deneault et al., 2019 [39]
3p	Deletion Size of CNV - not available in the paper	Keratinocytes	Patient: CytoTune-iPS Sendai Reprogramming Kit Controls: Constitutive Polycistronic Lentivirus Reprogramming Kit	Patient: 1 Controls: 3	Neurons (cortical neuron differentiation method based on dual SMAD inhibition)	Neural precursors Neural cells	Delay in expression of neuronal markers Dynamic imbalance in GABA/glutamate cell populations over time Enrichment of gene networks identified in autism post-mortem brains	Adhya et al., 2019 [38]
3p26.3	~ 1 Mb microduplication (begins approximately 600 kb upstream of the *CNTN6* gene and ends more than 50 kb downstream of its stop codon)	Fibroblasts	LeGO lentiviral vectors containing *OCT4*, *SOX2*, *C*-*MYC* and *KLF4*	Patient: 1 Controls: 2	Neurons (Ngn2 overexpression protocol) Neurons (through neural rosette stage)	Neural progenitors Layer 2/3 excitatory Cortical neurons	Neurons showed the characteristics of mature neurons based on the presence of neuronal markers and their electrophysiological activities	Gridina et al., 2018 [41]
de l[5](p14)	Microdeletion Size of CNV -not available in the paper	Peripheral blood mononuclear cells	CytoTune-iPS Sendai Reprogramming Kit	Patient: 1 Control: 1	**/**	**/**	/	Piovani et al., 2019 [42]
7q11.23	~ 1.6–1.8 duplication	Fibroblasts	Synthetic mRNAs encoding the *POU5F1* (*OCT4*), *SOX2*, *KLF4*, *LIN28A* and *MYC*	Patients: 2 Controls: 3	Dorsal telencephalic lineage Neural crest stem cells	Telencephalic neural progenitor cells Neural crest stem cells	Disruption of transcriptional circuits in disease-relevant pathways	Adamo et al., 2015 [43]
8p23.3	De novo 791 kb duplication/+ (chr8:704001-1535000dup) (primary genetic variant) Other ASD implicated variants detected in patient: - 8p22-p21.3 823 kb duplication/+ - RNF148 p.R225X/+ - CHD7 p.E1897K/+ - RAI1 p.G1864R/+	Fibroblasts	CytoTune-iPS Sendai Reprogramming Kit	Patient: 1 Controls: 11 (2 family controls—unaffected father and affected brother) (total in the study—controls: 11; ASD-affected: 14)	Neurons (NGN2 ectopic expression approach)	Glutamatergic neurons	No difference in weighted mean firing rate between patient and family controls	Deneault et al., 2019 [39]
8q21.12 - q21.13	Deletion Size of CNV - not available in the paper Additional findings detected in patient: 19:41759516 C>T	Keratinocytes	Patient: CytoTune-iPS Sendai Reprogramming Kit Controls: Constitutive Polycistronic Lentivirus Reprogramming Kit	Patient: 1 Controls: 3	Neurons (cortical neuron differentiation method based on dual SMAD inhibition)	Neural precursors Neural cells	Delay in expression of neuronal markers Dynamic imbalance in GABA/glutamate cell populations over time Enrichment of gene networks identified in autism post-mortem brains	Adhya et al., 2019 [38]
9q34.3	Mosaic 233 kb microdeletion (proximal breakpoint between exons 4 and 5 of the *EHMT1* gene and distal breakpoint between exons 10 and 11 of the *CACNA1B* gene)	Fibroblasts	Retroviral vectors expressing *OCT4*, *SOX2*, *KLF4* and *cMYC* CRISPR line was generated by nonintegrating Sendai virus	Patient: 1 (iPS clone harbouring the microdeletion as well as a control clone not carrying the microdeletion were selected) Controls: 2	Neurons (Ngn2 overexpression protocol)	Excitatory cortical layer 2/3 neurons	Reduced H3K9me2 immunoreactivity Network bursts is occurred at a lower frequency and with longer duration Longer inter-burst interval Smaller percentage of spikes occurring outside the network bursts Network burst activity strongly depends on NMDA receptor mediated transmission	Frega et al., 2019 [44]; Willemsen et al., 2011 [45]
11q22.1	Maternal 676 kb deletion/+ (chr11:99477401-100157000del)	Fibroblasts	CytoTune-iPS Sendai Reprogramming Kit	Patient: 1 Controls: 11 (1 family control) (total in the study: controls: 11; ASD-affected: 14)	Neurons (NGN2 ectopic expression approach)	Glutamatergic neurons	Increased neuronal activity	Deneault et al., 2019 [39]
Deletion in chromosome 14	4.8 kb deletion (chr14:39987476-39992327)	Fibroblasts	retroviruses containing *OCT4*, *SOX2*, *KLF4* and c-*MYC*	Patient: 1 Controls: (a) 2 unaffected, first-degree family members (mother, father) (b) PGP1-1 iPSC line (Ball et al., 2009) (c) K3 iPSC line (Si-Tayeb et al., 2010)	Telencephalic organoids	Radial glia Intermediate progenitors Neurons	Upregulation of genes involved in cell proliferation, neuronal differentiation and synaptic assembly Decrease in cell-cycle length in iPSCs and neuronal progenitors Increased neuronal differentiation and synaptic connections Increase in the number of inhibitory synapses Overproduction of GABAergic inhibitory neurons	Mariani et al., 2015 [46]; Abyzov et al., 2012 [47]
15q11.2	∼ 382 kb microdeletion between BP1 and BP2	Fibroblasts	Sendai virus (five constructs -TS7-*OCT3/4*, -*SOX2*, -*KLF4*, -c-*MYC* and GFP)	Patients 2 Control: 1	Neurons	Neural progenitor cells Neurons	Altered dendritic morphology	Das et al., 2015 [48]
15q11.2-q13.1	~ 5.57 Mb duplication (chr15:21,144,837-26,722,409)	Peripheral blood mononuclear cells	Episomal vectors (pCE-h*OCT3/4*, pCE-hSK, pCE-hUL, pCE-mp53DD and pCXB-EBNA1)	Patient: 1	/	/	/	Arioka et al., 2018 [49]
15q11- q13.1	Isodicentric and interstitial duplications of 15q11-q13	Fibroblasts Umbilical cord blood	Retroviral, lentiviral or episomal vectors encoding *OCT4*, *SOX2*, *KLF4*, *MYC* and *LIN28*	Patients: 4 (two individuals with isodicentric [15], one with a paternally inherited duplication of chromosome 15q11-q13.1 and one individual mosaic for a maternally inherited interstitial duplication of chromosome 15q11-q13.1) Control: 1	Neuron (embryoid body-based protocol or monolayer differentiation)	Vesicular glutamate transporter 1-positive excitatory neurons Glutamate decarboxylate 65-positive inhibitory Neurons	Downregulation of genes involved in neuron development Upregulation of genes involved in cell cycle and protein catabolic processes in isodicentric chromosome 15 neurons	Germain et al., 2014 [50]
15q13.3	Heterozygous 15q13.3 deletions and duplications Patient 1—BP4/BP5 duplication (2.1 Mb) (second hit CNVs detected - 6q21 duplication) Patient 2—BP4/BP5 deletion Patient 3—BP3/BP5 deletion (second hit CNVs detected -17q12 loss) Patient 4—BP3/BP5 duplication Patient 5—D-CHRNA7-LCR/BP5 duplication Patient 6—BP4/BP5 deletion	Fibroblasts	CytoTune-iPS Sendai Reprogramming Kit	Patients: 6 Controls: 3	Neural progenitor cells (NPCs) (dual SMAD inhibition protocol)	Cortical-like neural progenitor cells	α7 nicotinic acetylcholine receptor (α7 nAChR)-associated calcium flux was decreased in 15q13.3 deletion and duplication probands Increased gene expression of chaperones involved in folding, assembly and trafficking α7 nAChRs in 15q13.3 duplication NPCs Increased expression of a subset of ER stress markers in 15q13.3 duplication NPCs Decreased expression of JAK2 in both CNV groups	Gillentine et al., 2017 [51]
16p11.2	De novo 616 kb deletion/+ (chr16:29584000-30200000del)	Fibroblasts	Retroviruses expressing *OCT4/POU5F1*, *SOX2*, *KLF4* and *MYC* and lentiviral vector containing pluripotency reporter EOS-GFP/PuroR	Patient: 1 Controls: 11 (1 family control—unaffected father) (total in the study: Controls: 11; ASD-affected: 14)	Neurons (NGN2 ectopic expression approach)	Glutamatergic neurons	/	Deneault et al., 2019 [39]
16p11.2	Microduplication (1 patient) Microdeletion (3 patients) Size of CNV—not available in the paper	Fibroblasts	Episomal plasmids pCXLE-hOct3/4-shp53-F, pCXLE-hSox2-Klf4, pCXLE-hcmyc-Lin28	Patient: 4 Controls: 4	Neurons	Forebrain cortical neurons	16pdup neurons—reduced neuronal size and dendrite length, less complex dendritic arbors, reduced soma size, reduced synaptic density, increased synaptic strength and lower density of excitatory synapses 16pdel neurons—increased soma size and dendrite length, more extensive dendritic arbors, reduced synaptic density, increased synaptic strength, lower density of excitatory synapses, higher current needed to fire first action potential (AP) 16pdel neurons fired far fewer APs than the control and 16pdup neurons Reduced voltage responses of 16pdel neurons	Deshpande et al., 2017 [52]
16p13.11	Heterozygous 1.65 Mb microduplication (chr16: 14,892,975-16,544,033) (de novo loss-of-function variant in *TSC2* at 16:2115634:C/T)	Fibroblasts	Episomal—plasmids containing *Oct4*/shP53, *SOX2/Klf4* and *L*-*Myc*/*Lin28* (pCXLE-hSK and pCXLE-hUL) Control 5—retroviruses containing *Oct4*, *Sox2*, *Klf4* and c-*MYC*	Patient: 1 Controls: 5	Anterior neural precursor cells (NPCs) Cerebral organoids	Anterior neural precursor cells Cerebral organoids (neural precursor cells and neurons)	Reduced NPC proliferation Smaller organoids Organoids - Far fewer neuronal progenitor cell regions - Reduced numbers of total dividing neuronal progenitor cells - Altered planes of cell division Deficit in the NFκB p65 pathway in NPCs and cerebral organoids	Johnstone et al., 2019 [53]
17p13.3	Patient 1—4.5 Mb deletion Patient 2—5.7 Mb deletion Patient 3—2.7 Mb deletion	Fibroblasts	Episomal—plasmids encoding *OCT3/4*, *SOX2*, *KLF4*, L-*MYC*, *LIN28* and shRNA for *TP53*	Patients: 3 Controls: 3	Cerebral organoids	Cerebral organoids (neuroepithelial stem cells, neurons)	Smaller organoids Organoids - Increased apoptosis in cortical ventricular zone-like regions - Decreased vertical divisions - Defective neuronal migration - Increased abundance of deep-layer neurons Mitotic defect (delay in cell division) in outer radial glia cells	Bershteyn et al., 2017 [54]
22q13	Patient 1—871 kb microdeletion Patient 2—825 kb microdeletion	Fibroblasts	Retroviruses carrying *SOX2*, *OCT3/4*, c-*MYC*, *KLF4*	Patients: 2 Controls: H9-ESC line, IM23-9 and NH1-1 cell lines (Yazawa et al., 2011; Paşca et al., 2011)	Neurons	FoxG1/Pax6- positive telencephalic neuronal precursors Neurons	Reduced number of neurons Defects in excitatory synaptic transmission Reduced number of excitatory synapses Reduction in the expression of glutamate receptors Reduced level of SHANK3 protein expression	Shcheglovitov et al., 2013 [55]
22q13.33	Microdeletion in *SHANK3* gene Size of CNV—not available in the paper	Keratinocytes	Polycistronic lentiviral construct coexpressing *OCT4*, *SOX2*, *KLF4* and c-*MYC*	Patients: 2 Controls:3	Neurons (modified version of dual SMAD inhibition protocol)	Cortical and olfactory placodal neurons	Fewer puncta labelled with both presynaptic and postsynaptic markers Placodal neurons—smaller cell diameter, higher neurite length and the mean number of neurites Cortical neurons—shorter neurites Higher rate of formation of the primary neurite Lower rate of primary neurite elimination Higher rate of extension of primary neurite length Lower rate of primary neurite length retraction Reduced soma speed Slower growth	Kathuria et al., 2018 [56]; Cocks et al., 2014 [57]
Xp22.11	Patient 1—167 kb microdeletion that eliminates the promoters and first exons of PTCHD1 and PTCHD1-AS Patient 2—125 kb microdeletion that eliminates the conserved third exon of *PTCHD1*-*AS* and *DDX53*	Fibroblasts CD34+ blood cells	Fibroblasts—retrovirus vectors blood cells—Sendai virus	Patients: 2 Controls: 2 (unaffected mother of one patient and unaffected male)	Neurons	Neural progenitors cells Cortical neurons	Reduced miniature excitatory postsynaptic current frequency *N*-methyl-d-aspartate receptor hypofunction	Ross et al., 2019 [58]
Xq11.1	216.7 kb microdeletion (chrX.hg19:g.62856174_63072861) including the entire *CB* gene (*ARHGEF9*)	Fibroblasts	Retrovirus vectors containing the *OCT4*, c-*MYC*, *KLF4* and *SOX2*	Patient: 1 Controls: 2	Neurons	Neural progenitor cells Cortical neurons	Increases in mTORC1 signalling activation and translation initiation	Machado et al., 2016 [59]
Xq28	Patient 1—300 kb duplication (Xq28 (152.73–153.02 Mb)) Patient 2—15.25 Mb (Xq28 (139.33–154.58 Mb)) duplication Patient 3—500 kb (Xq28 (152.66–153.15 Mb)) duplication	Fibroblasts	pMXs retroviral vectors containing *OCT4*, *SOX2*, *KLF4* and C-*MYC*	Patient: 3 Controls: 2 healthy persons and BJ1 fibroblasts	Neurons	Forebrain progenitors Pyramidal neurons	Increased synaptogenesis and dendritic complexity Altered neuronal network synchronisation	Nageshappa et al., 2016 [60]

All NDD conditions are characterised by aberrant brain and cognitive function that arises during pre-natal or early post-natal life stages. Ultimately, these changes result in altered neuronal function and are strongly associated with synapse function and neuronal activity. However, genetic risk does not necessarily arise directly from mutation of synaptic protein-encoding genes and may also arise from deficits that occur in early neurodevelopment leading to abnormal neurogenesis or synaptogenesis. In the following section, we relate the aspects of ASD biology to the different modes of study available for patient iPSCs.

### Synapse biology and neuronal activity

Accumulating evidence indicates altered brain connectivity as a common feature across all psychiatric disorders, implying underlying abnormalities of the brain circuitry [61]. Analysis of neuronal connectivity via synapses in cultured neurons can be performed using immunocytochemistry of synaptic proteins to examine synaptic density, rabies virus techniques to identify which neurons are connected via synapses, and a range of electrophysiological techniques that allow the detection of postsynaptic currents and potentials [62].

Studies on the glutamatergic excitatory neuronal synapse from ASD patient iPSCs with a 22q13.3 deletion or de novo mutations of the synaptic protein Shank3, which is located within the CNV, showed that these neurons have significantly reduced numbers of synapses and a corresponding decrease in synaptic transmission. These deficits can be reversed by treatment with the insulin-related hormone IGF-1 [55, 63]. Consistent with synaptic transmission deficits, iPSC-derived neurons from 15q11-q13 duplication syndrome were shown to be associated with delayed action potential maturation and increased synaptic event frequency. iPSC-derived neurons from 16p11.2 deletion and duplication were again associated with a decreased number of synapses. However, in contrast to other reports, the amplitude of excitatory postsynaptic currents of 16p11.2-derived neurons was significantly higher suggesting that they have increased synaptic strength [52]. Investigation of patient iPSCs with the 9q34 del (Kleefstra Syndrome) indicated a network disruption that correlates with increased expression of the NMDA subunit GluN1 that can be reversed by the NMDA blocker MK-801 [44].

These observations suggest that altered brain activity associated with CNVs can be captured in vitro in iPSC culture, offering an excellent platform for pharmacology of human neuronal systems. However, they are not the only differences that can be observed using patient iPSCs.

### Gene regulation and neurodevelopment

iPSCs uniquely offer the means to follow neurodevelopment in vitro at the cellular level, providing a direct way to follow timing and fate during cell differentiation. This can be readily investigated by monitoring gene expression by RNA profiling techniques, such as RNA-seq, and assayed in conjunction with immunocytochemistry and cellomic approaches to localise changes to specific cells.

Studies of iPSCs derived from idiopathic ASD patients with no genetic or clinical stratification identified gene modules (sets of co-expressed genes), which are mis-regulated in the patient lines [64]. Affected modules, included those involved in synaptic transmission, correlated with altered neuronal network activity, as measured by multi-electrode array (MEA) and calcium signalling. A recent iPSC study focused on ASD patients who exhibit macrocephaly identified heterochronic disease-associated changes of gene expression and chromatin accessibility during their neuronal differentiation that could be reversed via expression of the neurogenic transcription factor *NGN2* [65].

Direct examples of a gene regulatory change due to a CNV are Kleefstra syndrome and 7q11.23 (WBS). Loci of both CNVs contain epigenetic regulators that repress transcription to ultimately lead to synaptic dysfunction, either through loss of the *EHMT1* gene, which generates the suppressive histone methylation H3K9me2 [66] in the case of Kleefstra syndrome or GTF21 in WBS [68]. GTF21 dosage imbalance is responsible for a large proportion of the transcriptional deregulation in WBS acting mainly as a transcriptional activator or cooperating with the H3K4 demethylase LSD1 in mediating transcriptional repression [43, 67]. The CNV 1q21.1 also contains a remodeller but its gene regulatory function has yet to be investigated. In addition to direct changes where CNV result in loss of a gene regulator, indirect changes can also lead to altered expression, for example the transcriptional profile changes associated with loss of *Shank3* [68]. RNA profiling methods and cell localisation approaches can be combined together using single-cell RNA-seq to observe altered expression as cells diverge into different cell states, as reported from WBS and NRXN-1 CNVs [40, 67].

### Neuro-inflammatory pathways

A third major biological pathway identified from GWAS is neuro-inflammation. Although this seems quite distinct from synaptic biology and neurodevelopment, in the context of the nervous system immunological pathways play a major part in shaping neuronal interactions and function. Elevated levels of TNF-α, IL-1β, IL-6 and IL-17 in the brains of ASD patients support the notion that dysregulated immunomodulatory pathways contribute towards pathology [69].

Post-mortem brain tissue from ASD patient has shown an increased microglial activation in the prefrontal cortex and microglia-specific gene expression has been found to be altered. Deficits in microglial activity were shown to reduce synaptic pruning leading to altered brain connectivity due to an accumulation of immature synapses [70]. It is not yet clear whether the increase in peripheral inflammation reported for 22q11, 16p11.2, 3q and 7q11.23 CNVs are reflected in microglial dysfunction, but loss of the *Cyfip1* gene present within the 15q11.2 CNV in mice leads to increased neurogenesis due to a failure of microglial-induced neuronal apoptosis [71]. Protocols for differentiation of microglia from iPSCs have been developed, and these microglia were shown to secrete pro-inflammatory cytokines upon stimulation. However, their effect on synaptic pruning and brain development has not been yet reported.

Although neuronal dysfunction receives most attention, iPSC-based studies have indicated dysfunction of glial cells. In neuron-astrocyte co-cultures, it was shown that ASD patient astrocytes can interfere with neuronal development, while control astrocytes could rescue changes in neuronal morphology and synaptogenesis in non-syndromic ASD cultures [72]. Increased levels of IL-6 in the ASD astrocytes were suggested to underlie astrocyte actions, in line with earlier studies showing involvement of immune system dysregulation in ASD [73]. Recent single-cell expression studies on iPSC-derived neural stem cell cultures also revealed that *NRXN1*-*α* deletions shift neural cell development into higher proportions of astroglial cells [40], thereby affecting functional maturation of neurons. In addition to astroglia, a recent study also indicated involvement of oligodendroglia in neuronal phenotypes associated with ASD, as shown by neuron-oligodendrocytes co-cultures generated from tuberous sclerosis complex (TSC) patient cells [74]. Whether the cellular changes in morphology are also linked to the ASD phenotypes is so far unclear but will continue to be an expanding area for CNV patient iPSC studies.

### Cortical abnormalities

It has been widely reported that brains of children with ASD have accelerated growth compared to non-patient controls of similar age, known as macrocephaly [75–78]. Magnetic resonance imaging (MRI) studies indicate wide-reaching phenotypic impact in some rare CNVs leading to substantial size and shape changes in the brain, e.g. 22q11 [79, 80], 7q11.23 deletion [81, 82], 15q11.2 [83–85] and 16p11.2 proximal [86–88] and distal CNVs [89], potentially underlining a neurodevelopmental component to the disorders. As such, a recent study using iPSCs from ASD patients with macrocephaly identified disease-associated changes at transcriptome and cellular levels that are present in a very early stage of neural stem cells and established an ASD specific gene signature. More importantly, overexpression of module-specific gene signatures in healthy cells was enough to recapitulate ASD disease-specific cellular phenotypes [65]. These findings demonstrate that there are key nodes within dysregulated gene networks that are related to ASD and may represent promising targets for therapeutic intervention.

The presence of additional numbers of neurons in the prefrontal cortex of the post-mortem brain from ASD patients complements clinical observations [75]. Furthermore, changes in neuronal density, neurogenesis and increased cortical thickness were found in subjects diagnosed with ASD [90, 91]. Using iPSCs, Deshpande et al. [52] assessed changes in brain growth phenotypes, such as cellular morphology in iPSC-based cortical cultures derived from 16p11.2 deletion and duplication patients. In line with clinical changes, neuronal cultures developed from 16p11.2 deletion iPSCs possessed increased soma size and dendritic length, while those from the duplication had reduced cell size and dendritic length. A further correlation between brain size and increased proliferation of neuronal progenitors has also been shown in iPSC-based cultures of individuals with idiopathic ASD [92].

Use of 3D brain organoid cultures have shown that changes in even a single gene, such as *FOXG1* [46] *and CHD8* [12], can lead to dysregulation of the cortical layer formation, cell migration and cell division and potentially increased differentiation GABAergic neurons. Organoid modelling 17p13.3 deletion (Miller-Dieker syndrome) demonstrated mitotic defects in the outer radial glia, cell migration and also an overproduction of GABAergic neurons [10, 54]. However, although the 17p13.3 deletion is associated with both ID and unprovoked seizures, it is the duplication that is associated with risk of ASD, and this has not been reported to have a deficit in GABAergic neuronal number. Furthermore, there is no consensus on whether ASD is associated with decrease or increased GABAergic neurons, even whether it is an excitatory/inhibitory imbalance per se, not its direction, that increase risk [93]. Organoids derived from CNV-associated patient offer good potential to resolve this question in future.

### White matter alterations

Emerging studies suggest that white matter pathology also contributes towards the pathogenesis of ASD. Patients show regional decrease of white matter and increase of the grey matter [94–96]. The presence of supernumerary neurons within the white matter of the subcortical region is a commonly observed anomaly of ASD. Additionally, white matter integrity as measured by fractional anisotropy seems to be altered in 22q11.2 deletion patients [97] and 16p11.2 deletion is also associated with decreased myelination in the subcortical region of the brain [98]. A recent animal study of Cyfip1 function in the corpus callosum showed that its deletion decreases the myelinating potential of oligodendrocytes [99]. Although it is still not understood what induces hypomyelination in the ASD, this may arise from deficits in oligodendrocyte progenitor cell differentiation or dysregulated apoptosis of mature oligodendrocytes leading to decreased neuronal myelination. This interaction can be modelled with patient iPSCs, and cells from ASD patients with loss of function *TSC*-*1* or -*2* mutations exhibit oligodendrocyte-neuronal signalling and altered cell differentiation [74]. Given the evidence from brain imaging and animal studies, future in vitro cell studies of iPSCs derived from patients harbouring pathogenic CNVs are likely to be very informative.

## Limitations

While the “What aspects of ASD can be modelled in iPSCs derived from CNV patients?” section outlines how in vitro cell cultures of iPSCs derived from patients harbouring pathogenic CNVs can capture disease-relevant features seen in the patient brain, we have to recognise a number of limitations and potential confounds.

1. Cellular reprograming can contribute to formation of de novo CNVs in pluripotent stem cells during early cell passage, although both the size and total number seems to decrease with subsequent passages possibly due to negative selection [100]. Such genomic instability presents a potential serious confound for studies of iPSCs derived from patients harbouring pathogenic CNVs and has been shown to have adverse effect on pluripotency, cell proliferation and differentiation [101–104], all features of the patient cell phenotype. Careful monitoring of the cell karyotype and regular array-based or WGS genotyping is required to eliminate any cell lines that have accumulated artefactual CNVs during their generation. It is also recommended that newly generated lines should be grown for a minimum of 10 passages to ensure genetic stability, coupled with genotyping and differentiation.
2. Gene expression analyses indicate that iPSC-derived neurons appear to be in a pre-natal state [105, 106] and several ageing-associated changes are erased during the reprogramming process [107]. This is a useful feature when using iPSCs for studying neurodevelopmental deficits. It does however mean that genome imprinting and other epigenetic changes associated with parental and environment interactions may be lost [108]. Future studies modelling ASD may benefit from monitoring changes in chromatin modification at imprinted and other loci.
3. Differentiation of human iPSCs extends over long culture times, taking up to 100 days or more to fully form functionally mature neurons, and even then, they often show characteristics of foetal embryonic neurons [109]. This prolonged process affords plenty of opportunities for small variations in culture conditions to introduce differences from one culture to the next. Direct comparisons of gene expression, by use of RT-qPCR or RNA-seq, and cell electrophysiology need to be made to ensure that cultures have reached the same level of maturity. Currently, whole-cell recordings of iPSC-derived neurons are routinely performed and provide accurate measurements of the intrinsic properties of these cells. Relevant parameters to study the degree of neuronal differentiation (such as stable resting membrane potential, input resistance, membrane capacitance and action potential characteristics) can be determined by measuring the voltage response of the cell to injected hyperpolarizing or depolarizing current pulses. Where fine comparisons are needed between patient and non-patient controls for different cell types, single-cell RNA-seq or even Patch-seq for neurons, which combines patch-clamp and RNA-seq [110], may be beneficial.
4. Finally, the in vitro culture conditions, whether 2D or 3D, employed for iPSC studies rely on the self-assembly of cell-cell contacts and synaptogenesis. Although this may model the dynamics of neurogenesis in the brain, it does not recapitulate the higher order organisation and circuitry seen in the human brain. This does currently limit the scope of iPSC work, but solutions are now in development, such as MEA analysis of 3D brain organoids [111].

## Future development

Current usage of iPSCs from ASD and other NDD patients with associated CNV is improving our understanding of disease mechanism at the neural cell level. Importantly as discussed in the “What aspects of ASD can be modelled in iPSCs derived from CNV patients?” section, the cell phenotypic data that can be gained from these studies do align with observations from patients and in the clinic. This demonstrates the utility of iPSCs derived from patients harbouring pathogenic CNVs. But how can this be developed further to enhance their future role for study of the origins of ASD and ultimately for development of new therapeutics?

**Fig. 1:**
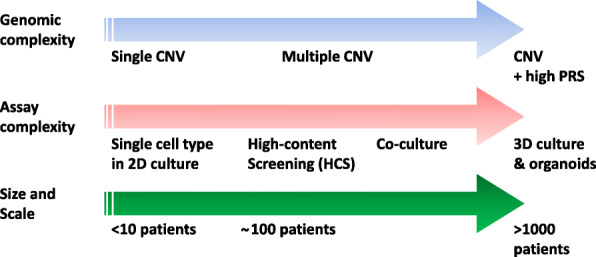
Three domains for future expansion of iPSC studies. Increases in the number of patient iPSCs within a study (from low to high); assay complexity (from single parameter of 2D cultures to complex, multi-parameteric, high content assays on co-cultures or in 3D structured and organoid conditions) and increases in cell genetic complexity (from single CNV (monogenic) to multiple CNV and increasing polygenic (PRS) genomic background)

We propose that the next generation of iPSC studies need to expand in three domains: genomic complexity, cell assay complexity and scale (Fig. 1). In combination, all three will build an enhanced cell-based platform for the study of ASD and other NDD in vitro and at the cellular level with increasing construct validity.

### Genomic complexity

In the simplest cases, CNV patients, in which only a single gene is deleted or duplicated and also exhibits high genetic penetrance, can approximate to a monogenic disorder. Such cases are seen for 9p34 (Kleefstra syndrome) and *NRXN1*-*α*, and a case can be made for *SHANK3* where individual gene mutations share the same clinical phenotype as the patient with 22q13.2 CNV. However, for most CNVs multiple candidate genes lie within the affected chromosome locus. To progress these cases, we need to establish the mechanism of disease on an individual CNV basis. In principle, this can be achieved by gene transcriptional analysis of the genes within the CNV and cross-reference to GWAS data across the range of NDDs and associated comorbidities. In practice, this can be challenging as multiple genes can often be expressed with patterns that vary from one cell type to another or developmental state; comparison of deletions and duplications at the same loci often do not show “mirror”, or reciprocal expression patterns and GWAS data for NDD is still not saturated for all possible loci.

A further confound is the suspicion that many large CNV clinical phenotypes may arise due to the effects of multiple genes within the locus. Here, patient iPSCs studies may help, as CRISPR-generated isogenic human iPSCs may be directly compared to patient-derived iPSCs. Current genome editing methodologies are already available to allow with the generation of multiple gene manipulations to model these possibilities [112].

Such comparisons can also address patient genetic complexity due to the presence of either multiple CNVs or possession of common variants that elevate the polygenic risk in the genome background. The key to these approaches however will be to have a complete analysis of genomes of patients harbouring pathogenic CNVs, especially if these can be combined with family studies. The most complex genetic scenario is if the structural variation at a CNV alters gene regulation via long-range changes in nuclear architecture or trans-acting regulatory RNA molecules. Again, this would synergise well with patient iPSC approaches via multi-omic methodologies to combine cell phenotyping with genome-wide transcriptional profiling, including miRNAs and lncRNAs, and chromatin interaction studies. Given that GWAS studies indicate that for NDDs 80% of disease-associated SNPs are not within protein-coding genes, such patient iPSC studies may prove a powerful stepping-stone to understanding the impact of the majority of SNPs on inherited ASD risk.

### Assay complexity

Although progress has been made, there is still much further work required to improve construct validity of iPSC-based NDD assays. Many neuronal and glial cell types can be generated using available in vitro differentiation protocols. However, cell type diversity is still missing, particularly for the GABAergic interneuron populations, such as the rapid firing PV+ interneurons, which play an important regulatory function and exhibit abnormal behaviour in NDD. Expression of certain combinations of transcription factors can rapidly induce homogeneous populations of glutamatergic, excitatory neurons in the case of *NGN2*, and GABAergic, inhibitory neurons by expression of *Ascl1* and *Dlx2* [113]. The expectation is that more transcription factor combinations will be established to enable a greater range of cell type to be generated. However, this approach is not without issues. Given the evidence that the neuronal developmental programme may be disrupted in ASD patient cells, accelerating their neural cell differentiation may bypass some aspects of the cell phenotype. Further work remains to compare iPSC-derived neurons using classical and induced methods to ensure that they accurately reflect those found in the human brain.

In addition to increased assay complexity through generation and co-culture of multiple neuronal and glial cell types, a second element is to create more structured cultures beyond simple growth and development on 2D surfaces. Any increase in cell culture complexity needs to be standardised and deliver robust readouts. The development of 3D brain organoids aims to address this need; however, individual organoids vary considerably in structure and standardised methods of comparison still need to be developed and adopted. A compromise position that has a high degree of controllability, and hence potential for standardisation, is to use 2D cultured cells presented in a layered configuration. Simple examples of this are “sandwich” cultures where neurons and astrocytes are grown on coverslips and dishes as 2D monolayers and then placed together [114]. More sophisticated structured culturing methods are also being developed using 3D bioprinting [115] to build layered cultures where different cells types are set in layers of matrix creating flat interfaces between different cell types.

Finally, complexity can be increased via high content, multiparametric data collection. This can be achieved through increasing the number of parameters recorded for individual assay modalities, such as cellomic approaches with automated cell microscopy, or collection of MEA functional data, all of which are compatible with multi-well formats. Alternatively, it is possible to combine modalities together, so that electrophysiological techniques can be combined with imaging of cell and neuronal gene expression profiles in iPSCs to pinpoint exactly how patient cells differ from non-patient controls. This also requires creation of comparative databases that bring together morphological, genetic, gene expression, biophysical and electrophysiological properties across the neuro-differentiation time course. An exemplar is the Neuroelectro project that aims to compile and organise published data about electrical properties of neurons [116]. This is currently oriented to improve neuronal classification for animal models but could form the basis for a human iPSC platform.

### Size and scale

The third dimension is to expand the number of patient iPSCs within experiments, increasing the size of studies from less than 10 patients to many 100s if not 1000s of patients. New automation technologies, such as a fully automated robotic cell reprogramming system [117], are needed to allow the generation of large cohorts of patient-specific iPSCs. These need to be differentiated in parallel with robust and standardised protocols, which would include internal monitoring of the differentiation state.

Many of these issues can be solved by developing higher throughput and analysis culture techniques; however, the greatest limitation is the identification of sufficient numbers of CNV patients. The need to establish large patient cohorts and share data from these rare individuals in the population will be a strong driver for future international collaboration. Research networks, such as the MINDDS COST Action (CA16210) [118], are currently aiming to open up access to larger scale patient-based studies, including those based on iPSCs. As this endeavour grows, there will be the necessity to analyse, collate and share data across multiple sites. The solutions to these problems are already being developed, and projects such as RD-connect [119] may produce the platforms for effective data and resource sharing.

## Conclusions

Here, we have considered the need, current state and future of the utility of iPSC-based models of ASD derived from patients with associated CNVs. We have demonstrated that current evidence is accumulating for construct validity between the biological processes that can be studied at the cellular level and clinical observations on patients. We have also discussed the necessity to probe background genetics for iPSC studies of CNV patients, and the opportunity that their cell phenotyping offers for future research to resolve the biological effects of common variants on increased disease risk. Finally, we have considered the future of CNV patient-based cell platforms expanded into the three domains of increased genomic complexity, cell assay complexity and patient population size. While challenging, the expansion of CNV-focused iPSC investigations is feasible and the need to create larger patient iPSC panels will be a driver for future research. This global enterprise will create a unique cell interface to connect ASD risk genetics and clinical phenotype that accelerates the development of personalised medicine.

## Supplementary information

**Additional file 1: Supplementary Table 2a.** iPSCs generated from patients carrying CNVs and were diagnosed with ASD.

**Additional file 2: Supplementary Table 2b.** iPSCs generated from patients not diagnosed with ASD but carrying CNVs which might be associated with NDD.

## Data Availability

Not applicable

## Abbreviations

NDD: Neurodevelopmental disorders
ASD: Autism spectrum disorders
ID: Intellectual disability
iPSCs: Induced pluripotent stem cells
SNVs: Single nucleotide variants
CNVs: Copy number variants
WBS: Williams-Beuren syndrome
PMDS: Phelan-McDermid syndrome
miRNA: MicroRNA
lncRNA: Long non-coding RNA
ADHD: Attention-deficit/hyperactivity disorders
MEAs: Multi-electrode arrays
