# Supplementary material for: Copy number variants (CNVs): a powerful tool for iPSC-based modelling of ASD
Source: Mol Autism. 2020 Jun 1;11:42. doi: 10.1186/s13229-020-00343-4 (PMC7268297; doi:10.1186/s13229-020-00343-4)
Supplement: Supplementary file 1 — Additional file 1: Supplementary Table 2a. iPSCs generated from patients carrying CNVs and were diagnosed with ASD. [file 13229_2020_343_MOESM1_ESM.docx]

**Supplementary Table 2a. iPSCs generated from patients carrying CNVs and were diagnosed with ASD**

| **CNV** | **Type and size of CNV** | **Source** | **Reprogramming** | **Number of patients and healthy controls** | **Differentiation protocol** | **Neuronal cell types** | **Time from taken the somatic cells to phenotype neurons** | **Associated cellular phenotype** | **Validation and quality control of iPSCs** | **Isogenic**  **cell lines** | **Ref** |
| --- | --- | --- | --- | --- | --- | --- | --- | --- | --- | --- | --- |
| 1p21.3 | deletion  size of CNV - Not available in the paper | keratinocytes | patient: CytoTune-iPS Sendai Reprogramming Kit  controls: Constitutive Polycistronic Lentivirus Reprogramming Kit | patient: 1  controls: 3    (2 clones from each individual) | neurons (cortical neuron differentiation method based on dual SMAD inhibition ([1](#_ENREF_1))) | neural precursors  neural cells | from keratinocytes to iPSCs - not available in the paper  from iPSCs to early neural precursors – 8 days  from iPSCs to late neural precursors – 21 days  from iPSCs to cortical neural cells – 35 days | delay in expression of neuronal markers and dynamic imbalance in  GABA/glutamate cell populations over time in patient precursor cells compared to control precursor cells  higher expression of CD44 in patient neural cells compared to  control neural cells | expression of pluripotency markers (NANOG and TRA-1-81)  cytoSNP | No | ([2](#_ENREF_2)) |
| 1p33 | 323 kb deletion/+  (chr1:49894000-50224000del) (primary genetic variant)  other ASC implicated variants detected in patient:  - 2q21.1 516kb duplication/+  - HTR3A p.G148X/+ | fibroblasts | CytoTune-iPS Sendai Reprogramming Kit | patient: 1  controls: 11  (total in the study - controls: 11; ASC-affected: 14)  two iPSCs clones from each participant were selected for neuronal differentiation and phenotyping experiments | neurons (NGN2 ectopic expression approach ([3](#_ENREF_3))) | glutamatergic neurons | from fibroblasts to iPSCs - not available in the paper  from iPSC to neurons -1 week | at weeks 5 and 7 post-NGN2-induction weighted mean firing rate is reduced in patient cells compared to pool of ‘all controls | karyotype analysis  expression of pluripotency markers (SSEA4, Tra-1-60, OCT4, NANOG)  differentiation potential into three germ layer cells  STR analysis  mycoplasma testing | No | ([4](#_ENREF_4)) |
| 2p16.3 | bi-allelic *NRXN1-α* deletion  - paternal deletion: exon 1-5, ~ 0.4 kb  - maternal deletion: exon 1-5, ~ 0.18 kb | fibroblasts | CytoTune-iPS reprogramming kit | patient: 1  controls: 4 | neuroepithelial stem cells ([5](#_ENREF_5))  neurons (modified dual SMAD inhibition protocol ([6](#_ENREF_6))) | neuroepithelial stem cells (NESs)  neurons | from iPSCs to neural stem cells – 12 days  from iPSCs to neurons - 28, 49 and 70–75 days | complete absence of *NRXN1-α* expression during early development in patient cells  no difference in neural stem cell markers in patient compared to controls  slower proliferation rate in *NRXN1-α* del NES cells  no obvious morphological differences between control cells and *NRXN1-α* del cells at NES and differentiation (after 28 days) stage  contribution in cell types:  a) *NRXN1-α* del cells: radial glia-like cells, immature neurons and astroglia  b) control cells: neural stem cells, intermediate progenitors and neurons  *NRXN1-α* del NES cells differentiated towards neuronal (~50%) and non-neuronal cells (~50%) after 28 days of differentiation  slower amplitude and rise time in calcium signaling after 28 and 49 days of differentiation in *NRXN1-α* del cells  no mature neurons in *NRXN1-α* del cells after 70-75 days of differentiation  expression of cell adhesion genes (*CHL1* and *CNTN4*) is impaired in *NRXN1-α* del cells | morphology  karyotype analysis  expression of pluripotency markers (OCT4 and NANOG)  clearance of reprogramming vectors  microarray transcriptomes | No | ([7](#_ENREF_7)) |
| 2p16.3 | deletion  size of CNV - not available in the paper | keratinocytes | patients: CytoTune-iPS Sendai Reprogramming Kit  controls: Constitutive Polycistronic Lentivirus Reprogramming Kit | patients: 2  controls: 3  (2 clones from each individual) | neurons (cortical neuron differentiation method based on dual SMAD inhibition ([1](#_ENREF_1))) | neural precursors  neural cells | from keratinocytes to iPSCs - not available in the paper  from iPSCs to early neural precursors – 8 days  from iPSCs to late neural precursors – 21 days  from iPSCs to cortical neural cells – 35 days | delay in expression of neuronal markers and dynamic imbalance in  GABA/glutamate cell populations over time in patient precursor cells compared to control precursor cells  higher expression of CD44 in patient neural cells compared to  control neural cells | expression of pluripotency markers (NANOG and TRA-1-81)  cytoSNP | No | ([2](#_ENREF_2)) |
| 2p16.3 | *de novo* 430 kb deletion/+  (chr2:50567944-51057790del) | fibroblasts | retroviruses expressing *OCT4/POU5F1*, *SOX2*, *KLF4* and *MYC* and lentiviral vector containing pluripotency reporter EOS-GFP/PuroR | patient: 1  controls: 11  (total in the study - controls: 11; ASC-affected: 14)  two iPSCs clones from each participant were selected for neuronal differentiation and phenotyping experiments | neurons (NGN2 ectopic expression approach ([3](#_ENREF_3))) | glutamatergic neurons | from fibroblasts to iPSCs - not available in the paper  from iPSC to neurons - 1 week | no differences in weighted mean firing rate between patient and pool of ‘all controls | karyotype analysis  expression of pluripotency markers (SSEA4, Tra-1-60, OCT4, NANOG)  differentiation potential into three germ layer cells  STR analysis  mycoplasma testing | No | ([4](#_ENREF_4)) |
| 3p | deletion  size of CNV - not available in the paper | keratinocytes | patient: CytoTune-iPS Sendai Reprogramming Kit  controls: Constitutive Polycistronic Lentivirus Reprogramming Kit | patient: 1  controls: 3  (2 clones from each individual) | neurons (cortical neuron differentiation method based on dual SMAD inhibition ([1](#_ENREF_1))) | neural precursors  neural cells | from keratinocytes to iPSCs - not available in the paper  from iPSCs to early neural precursors – 8 days  from iPSCs to late neural precursors – 21 days  from iPSCs to cortical neural cells – 35 days | delay in expression of neuronal markers and dynamic imbalance in  GABA/glutamate cell populations over time in patient precursor cells, compared to control precursor cells  higher expression of CD44 in patient neural cells compared to  control neural cells | expression of pluripotency markers (NANOG and TRA-1-81)  cytoSNP | No | ([2](#_ENREF_2)) |
| del(5)(p14) | microdeletion  size of CNV -not available in the paper | peripheral blood mononuclear  cells | CytoTune-iPS Sendai Reprogramming Kit | patient: 1  control: 1 | **/** | **/** | from peripheral blood mononuclear cells infection to picking iPSCs colonies ~ 20 days | **/** | morphology  karyotype analysis  STR analysis  expression of  pluripotent transcription factors  differentiation capacity in three germ layers  mycoplasma testing  clearance of reprogramming vectors | No | ([8](#_ENREF_8)) |
| 7q11.23 | ~1.6-1.8 duplication | fibroblasts | synthetic mRNAs encoding *POU5F1* (*OCT4*), *SOX2*, *KLF4*, *LIN28A* and *MYC* | patients: 2 (6 iPSC lines)  controls: 3 (6 iPSC lines) | dorsal telencephalic lineage ([1](#_ENREF_1), [9](#_ENREF_9))  neural crest stem cells ([10](#_ENREF_10)) | telencephalic neural progenitor cells  neural crest stem cells | from fibroblasts to iPSCs – 16-20 days  from iPSCs to neural crest stem cells - ~15 days  from iPSCs to telencephalic neural progenitor cells - 32 days | transcriptional dysregulation in disease-relevant pathways | morphology  expression  of pluripotency markers  teratoma assay  analysis of genomic integrity by high-density CytoScan array | No | ([11](#_ENREF_11)) |
| 8p23.3 | *de novo* 791 kb duplication/+  (chr8:704001-1535000dup) (primary genetic variant)  other ASC implicated variants detected in patient:  - 8p22-p21.3 823 kb duplication/+  - RNF148 p.R225X/+  - CHD7 p.E1897K/+  - RAI1 p.G1864R/+ | fibroblasts | CytoTune-iPS Sendai Reprogramming Kit | patient: 1  controls: 11  2 family controls:  - unaffected father: primary genetic variant(s): None;  other ASC implicated variants:  8p22-p21.3 823kb duplication/+  RNF148 p.R225X/+  CHD7 p.E1897K/+)  - affected brother: primary genetic variant(s): None;  other ASC implicated variants:  8p22-p21.3 823kb duplication/+  RNF148 p.R225X/+  CHD7 p.E1897K/+  RAI1 p.G1864R/+))  (total in the study - controls: 11; ASC-affected: 14)  two iPSCs clones from each participant were selected for neuronal differentiation and phenotyping experiments | neurons (NGN2 ectopic expression approach ([3](#_ENREF_3))) | glutamatergic neurons | from fibroblasts to iPSCs - not available in the paper  from iPSC to neurons - 1 week | no difference in weighted mean firing rate between patient and family controls | karyotype analysis  expression of pluripotency markers (SSEA4, Tra-1-60, OCT4, NANOG)  differentiation potential into three germ layer cells  STR analysis  mycoplasma testing | No | ([4](#_ENREF_4)) |
| 8q21.12 -  q21.13 | deletion  size of CNV - not available in the paper  additional findings detected in patient: 19:41759516 C>T | keratinocytes | patient: CytoTune-iPS Sendai Reprogramming Kit  controls: Constitutive Polycistronic Lentivirus Reprogramming Kit | patient: 1  controls: 3  (2 clones from each individual) | neurons (cortical neuron differentiation method based on dual SMAD inhibition ([1](#_ENREF_1))) | neural precursors  neural cells | from keratinocytes to iPSCs - not available in the paper  from iPSCs to early neural precursors – 8 days  from iPSCs to late neural precursors – 21 days  from iPSCs to cortical neural cells – 35 days | delay in expression of neuronal markers and dynamic imbalance in  GABA/glutamate cell populations over time in patient precursor cells, compared to control precursor cells  higher expression of CD44 in patient neural cells compared to  control neural cells | expression of pluripotency marker (NANOG and TRA-1-81)  cytoSNP | No | ([2](#_ENREF_2)) |
| 9q34.3 | mosaic 233 kb microdeletion (proximal breakpoint between exons 4 and 5 of *EHMT1* gene and distal breakpoint between exons 10 and 11 of the *CACNA1B* gene) | fibroblasts | retroviral vectors expressing *OCT4*, *SOX2*, *KLF4* and c*MYC*  CRISPR line was generated by nonintegrating Sendai virus | patient: 1 (iPS clone harboring the microdeletion and control clone not  carrying the microdeletion were selected)  controls: 2  CRISPR/Cas9 technology was used to create a heterozygous *EHMT1* mutation in a iPS cell line derived from a healthy 51-year-old male | neurons (Ngn2 overexpression protocol ([3](#_ENREF_3))) | excitatory cortical layer 2/3 neurons | from iPSCs to neurons - 21 days | patient neurons showed reduced H3K9me2  immunoreactivity  21 days after the start of  differentiation no significant differences was observed between  control and patient neurons in neuronal somatodendritic morphology  in neurons obtained from the patient:  a) network bursts is occurred at a lower frequency and with longer duration  b) inter-burst interval was longer compared to control  c) spike organization is differed from control  d) network burst activity is strongly dependent on NMDAR mediated transmission, in contrast to control networks, where network bursts are mainly dependent  on AMPAR-mediated transmission | analysis of genomic integrity by SNP arrays  expression of the pluripotency markers (OCT4, TRA-1-81, NANOG, LIN28, SOX2,SSEA4) | Yes | ([12](#_ENREF_12), [13](#_ENREF_13)) |
| 11q22.1 | maternal 676 kb deletion/+  (chr11:99477401-100157000del) | fibroblasts | CytoTune-iPS Sendai Reprogramming Kit | patient: 1  controls: 11  (1 family control)  (total in the study:  controls: 11; ASC-affected: 14)  two iPSCs clones from each participant were selected for neuronal differentiation and phenotyping experiments | neurons (NGN2 ectopic expression approach ([3](#_ENREF_3))) | glutamatergic neurons | from fibroblasts to iPSCs - not available in the paper  from iPSC to neurons - 1 week | Increased neuronal activity in glutamatergic neurons deficient in one copy of *CNTN5* at week 6 post-NGN2-induction, compared with familial control | karyotype analysis  expression of pluripotency markers (SSEA4, Tra-1-60, OCT4, NANOG)  differentiation potential into three germ layer cells  STR analysis  mycoplasma testing | - in iPSC line, generated from a non-ASC and non-carrier individual, set of nonsense mutations was introduced to knock out the expression of *CNTN5* | ([4](#_ENREF_4)) |
| Deletion in chromosome 14 | 4.8 kb deletion (chr14:39987476-39992327) | fibroblasts | retroviruses containing  *OCT4*, *SOX2*, *KLF4* and c-*MYC* | patient: 1 (3 iPSC lines)  controls:  a) 2 unaffected, first-degree family members (mother, father) (3 iPSC lines/ individual)  b) PGP1-1 iPSC line ([14](#_ENREF_14))  c) K3 iPSC line ([15](#_ENREF_15)) | telencephalic organoids ([16](#_ENREF_16)) | radial glia  intermediate progenitors  neurons | from retroviral transduction to iPSCs colony picking up – 30 days  from iPSCs to beginning of terminal neuronal differentiation phase  – 11(13) days; after that four to five weeks under conditions favoring terminal differentiation | upregulation of genes involved in cell proliferation, neuronal differentiation and synaptic assembly in patient cells  decrease in cell-cycle length in patient iPSCs and neuronal progenitors  increased neuronal differentiation and synaptic connections in patient neurons  increase in the number of inhibitory synapses in patient neurons  overproduction of  GABAergic inhibitory neurons in patient neurons | morphology  expression of  pluripotency factors (NANOG, TRA1-60, SSEA-4, OCT4, SOX2)  gene expression analyses (RT-PCR, microarrays,  complete transcriptome)  analysis of demethylation of canonical pluripotency factor promoters  analysis of differentiation potential | No | ([17](#_ENREF_17), [18](#_ENREF_18)) |
| 15q13.3 | heterozygous  15q13.3 deletions and duplications  patient 1- BP4/BP5 duplication (2.1 Mb) (second hit CNVs detected - 6q21 duplication)  patient 2 – BP4/BP5 deletion  patient 3 - BP3/BP5 deletion (second hit CNVs detected -17q12 loss) | fibroblasts | CytoTune-iPS Sendai Reprogramming Kit | patients: 3  controls: 3  (2-3 iPSC clones per individual) | neural progenitor cells (dual SMAD inhibition protocol ([6](#_ENREF_6), [19](#_ENREF_19))) | cortical-like neural progenitor cells | from fibroblast to iPSCs colonies- ~ 21 days  from iPSCs to NPCs - 12 days | α7 nicotinic acetylcholine receptor (α7 nAChR)- associated calcium flux was decreased in 15q13.3 deletion and duplication probands  gene expression of chaperones involved in folding, assembly and trafficking α7 nAChRs was increased in 15q13.3 duplication NPCs  expression of a subset of ER stress markers was increased in 15q13.3 duplication NPCs  decreased expression of JAK2 is observed in both CNV groups | karyotype analysis  expression of pluripotency markers (OCT4, SSEA-4, SOX2) | Tai et al. generated 15q13.3 microduplication and microdeletion (2 Mb) via CRISPR/Cas9 ([20](#_ENREF_20)) | ([21](#_ENREF_21)) |
| 16p11.2 | *de novo* 616 kb deletion/+  (chr16:29584000-30200000del) | fibroblasts | retroviruses expressing *OCT4/POU5F1*, *SOX2*, *KLF4* and *MYC*, and lentiviral vector containing pluripotency reporter EOS-GFP/PuroR | patient: 1  controls: 11  (1 family control-  - unaffected father)  (total in the study: controls: 11; ASC-affected: 14)  two iPSCs clones from each participant were selected for neuronal differentiation and phenotyping experiments | neurons (NGN2 ectopic expression approach ([3](#_ENREF_3))) | glutamatergic neurons | from fibroblasts to iPSCs - not available in the paper  from iPSC to neurons - 1 week | / | karyotype analysis  expression of pluripotency markers (SSEA4, Tra-1-60, OCT4, NANOG)  differentiation potential into three germ layer cells  STR analysis  mycoplasma testing | No  Note: family control was subjected to CRISPR gene editing to knock out 14 ASC-risk genes (*ANKRD11*,  *AUTS2*,  *ATRX*,  *CHD8*,  *AFF2/FMR2*, *CAPRIN1*,  *CACNA1C*, *KCNQ2*, *SCN2A*, *ASTN2*,  *DLGAP2*, *CNTNAP2*,  *ANOS1*,  *TENM1*) ([22](#_ENREF_22)) | ([4](#_ENREF_4)) |
| 16p11.2 | microduplication (1 patient)  microdeletion (3 patients)  size of CNV - not available in the paper | fibroblasts | episomal plasmids pCXLE-hOct3/4-shp53-F, pCXLE-hSox2-Klf4, pCXLE-hcmyc-Lin28 | patient: 4  controls: 4  2–3 iPSC clones were analyzed per individual | neurons ([23](#_ENREF_23)) | forebrain cortical neurons | from fibroblast to iPSCs – 2 weeks  from iPSCs to neural  progenitor cells (NPCs) – 25 days  from iPSCs to long-term neuronal maturation - up to 14 weeks | proliferation rate of 16pdup and 16pdel NPCs was unchanged compared with controls  16pdup neurons - reduced neuronal size and dendrite length, less complex dendritic arbors, reduced soma size, reduced synaptic density, increased synaptic strength and lower density of excitatory synapses  16pdel neurons - increased soma size and dendrite length, more extensive dendritic arbors, reduced synaptic density, increased synaptic strength, lower density of excitatory synapses, higher current needed to fire first action potential  16pdel neurons fired far fewer APs than the control and 16pdup neurons  the voltage responses of 16pdel neurons were greatly reduced compared with control neurons | expression of pluripotency markers (NANOG, OCT4, SOX2, SSEA-4)  analysis of differentiation potential  array CGH – Illumina CytoSNP 850K array  analysis of episomal transgene expression | Tai et al. generated 16p11.2 microduplication and microdeletion (740 kb) via CRISPR/Cas9 ([20](#_ENREF_20)) | ([24](#_ENREF_24)) |
| 16p13.11 | Heterozygous 1.65 Mb microduplication (chr16: 14,892,975-16,544,033)  (de novo loss-of-function variant in TSC2 at 16:2115634:C/T) | fibroblasts | episomal - plasmids containing Oct4/shP53, SOX2/Klf4 and L-Myc/Lin28 (pCXLE-hSK and pCXLE-hUL)  control 5 - retroviruses containing *OCT4*, *SOX2*, *KLF4* and c-*MYC* | patient: 1 (2-3 iPSC clones per individual)  controls: 5 (1 or 2 clones per control) | anterior neural precursor cells (aNPCs) ([25](#_ENREF_25), [26](#_ENREF_26))  cerebral organoids ([27](#_ENREF_27), [28](#_ENREF_28)) | anterior neural precursor cells (aNPCs)  cerebral organoids (NPCs and neurons) | from fibroblasts to iPSCs - 21-25 days  from iPSCs to aNPCs - ~ 1 month  from aNPCs to cortical neuronal cultures - 1 month  from iPSCs to cerebral organoids - ~ 1 month | reduced patient NPC proliferation  organoids are smaller in patient compared to control  far fewer neuronal progenitor cell regions and reduced numbers of total dividing neuronal progenitor cells in patient cerebral organoids compared to controls  patient cerebral organoids display altered planes of cell division  deficit in the NFκB p65 pathway in patient derived NPCs and cerebral organoids | karyotype analysis  analysis of differentiation potential  expression of pluripotency markers (Tra-160, OCT3/4, SOX-2, NANOG)  microarray karyotype analysis (Affymetrix 750K Cytoscan array)  analysis of episomal transgene expression  mycoplasma testing | No | ([29](#_ENREF_29)) |
| 22q13 | patient 1- 871 kb microdeletion  patient 2 – 825 kb microdeletion | fibroblasts | retroviruses carrying *SOX2*, *OCT3/4*, c-*MYC*, *KLF4* | patients: 2 (patient 1 - 4 iPS cell lines; patient 2 – 2 iPS cell lines)  controls: H9-ESC line, IM23-9 and NH1-1 cell lines ([30](#_ENREF_30), [31](#_ENREF_31)) | neurons ([6](#_ENREF_6), [32](#_ENREF_32)) | FoxG1/Pax6- positive telencephalic neuronal precursors  neurons | from cultivated fibroblast to iPSCs – 21-30 days after first infection  from iPSCs to telencephalic neuronal precursors – 31-40 days  from telencephalic neuronal precursors to neurons – 2-3 weeks | patient neurons:  - reduced number of neurons  - defects in excitatory synaptic transmission  -reduced number of excitatory synapses  - reduction in the expression of glutamate receptors  - reduced level of SHANK3 protein expression | morphology  expression of pluripotency markers (NANOG, TRA-2-49/6E)  SKY analysis  teratoma formation assay  expression of exogenous transcription factor (SOX2, OCT3/4, c-MYC, KLF4) | No | ([33](#_ENREF_33)) |
| 22q13.33 | microdeletion in *SHANK3*gene  size of CNV - Not available in the paper | keratinocytes | polycistronic lentiviral construct coexpressing *OCT4*, *SOX2*, *KLF4* and c-*MYC* | patients: 2  controls:3 | neurons (modified version of dual SMAD inhibition protocol ([1](#_ENREF_1))) | cortical and  olfactory placodal neurons | derivation of keratinocytes from scalp hair - ~ 12 days  from keratinocytes to iPSCs colonies - ~ 21 days  from iPSCs to neural progenitors – ~ 12 days  from iPSCs to doublecortin positive neurons – ~ 26 days  from iPSCs to synaptically active neurons – ~70 days | fewer puncta labeled with both presynaptic and postsynaptic markers in the patient neurons compared with control  cell diameter is smaller in the patient placodal neurons while the neurite length and the mean number of neurites is higher  no differences between the control and patient cortical neurons in soma area and number of primary neurites per neuron  patient cortical neurons have shorter neurites than the controls  rate of formation of the primary neurite is higher in patient neurons than in controls, whereas the rate of primary neurite elimination is lower  rate of extension of primary neurite length is higher in patient neurons, whereas the rate of primary neurite length retraction is lower  patient neurons have reduced soma speed compared with control  patient cells grew more slowly than controls | analysis of pluripotency markers (Oct4, Nanog, SSEA4 and Tra1-81) expression and alkaline phosphatase enzymatic activity  differentiation potential into three germ layers  ‘PluriTest’- genome wide gene expression analysis ( Illumina HT12v4 beadchips)  karyotype analysis | human ES line is engineered to carry one or two copies of *SHANK3* mutation discovered in ASC | ([34](#_ENREF_34), [35](#_ENREF_35)) |
| Xp22.11 | patient 1 - 167 kb microdeletion  that eliminates the promoters and first exons of *PTCHD1* and *PTCHD1-AS*  patient 2 - 125 kb microdeletion that eliminates the conserved third exon of *PTCHD1-AS* and *DDX53* | fibroblasts  CD34+ blood cells | fibroblasts - retrovirus vectors ([36](#_ENREF_36))  blood cells - Sendai virus | patients: 2  controls: 2 (unaffected mother of one patient and unaffected male) | neurons ([37](#_ENREF_37), [38](#_ENREF_38)) | neural progenitors cells  cortical neurons | from somatic cells to iPSCs - ~ 1 month  from iPSCs to neural rosettes – 1 week  for alpha-amino-3-hydroxy-5-methyl-4-isoxazole propionic acid receptor (AMPAR)–miniature excitatory postsynaptic current (mEPSC) recordings - 13 to 17 weeks | patient neurons exhibited reduced miniature excitatory postsynaptic current frequency and N-methyl-D-aspartate receptor hypofunction | expression of pluripotency markers (OCT4, SSEA4)  differentiation potential into the three germ layers  teratoma formation assays  karyotype analysis  CytoScan HD whole genome single nucleotide polymorphism arrays  analysis of X chromosome inactivation | Yes; iPSCs from the unaffected unrelated male were used for genome editing (CRISPR)/Cas9) to delete *PTCHD1-AS*  exon 3 | ([39](#_ENREF_39)) |
| Xq11.1 | 216.7 kb microdeletion (chrX.hg19:g.  62856174_63072861) including the entire *CB* gene (*ARHGEF9*) | fibroblasts | retrovirus vectors containing the *OCT4*, c-*MYC*, *KLF4* and *SOX2* | patient: 1  controls: 2  (at least two iPSC clones were obtained from each individual) | neurons ([40](#_ENREF_40)) | neural progenitor cells  cortical neurons | / | not major differences in reprogramming and differentiation capacities between patient and control cells  increases in mTORC1 signaling activation and translation initiation in patient NPCs compared to control | analysis of aneuploidies using multiplex ligation-dependent probe amplification subtelomeric kits  expression of pluripotent markers (Lin28, Nanog, Sox2, Oct4) | No | ([41](#_ENREF_41)) |
| Xq28 | 500 kb duplication | fibroblasts | pMXs retroviral vectors containing *OCT4*, *SOX2*, *KLF4* and C-*MYC* | patients: 1  controls: 2 healthy persons and BJ1 fibroblasts | neurons ([42](#_ENREF_42)) | forebrain progenitors  pyramidal neurons | from cultivated fibroblast to iPSCs –3-4 weeks  from iPSCs to neurons – 30-60 days | increase synaptogenesis and dendritic complexity in patient cortical neurons  neuronal network synchronization is altered in patient-derived neurons | expression of pluripotent genes  analysis of potency to generate three germ layers  teratoma formation assay  array CGH  karyotype analysis | No | ([43](#_ENREF_43)) |

**References**

1. Shi Y, Kirwan P, Livesey FJ. Directed differentiation of human pluripotent stem cells to cerebral cortex neurons and neural networks. Nature protocols. 2012;7(10):1836-46.

2. Adhya D, Swarup V, Nagy R, Shum C, Nowosiad P, Jozwik KM, et al. Atypical neurogenesis and excitatory-inhibitory progenitor generation in induced pluripotent stem cell (iPSC) from autistic individuals. bioRxiv. 2019:349415.

3. Zhang Y, Pak C, Han Y, Ahlenius H, Zhang Z, Chanda S, et al. Rapid single-step induction of functional neurons from human pluripotent stem cells. Neuron. 2013;78(5):785-98.

4. Deneault E, Faheem M, White SH, Rodrigues DC, Sun S, Wei W, et al. CNTN5(-)(/+)or EHMT2(-)(/+)human iPSC-derived neurons from individuals with autism develop hyperactive neuronal networks. eLife. 2019;8.

5. Falk A, Koch P, Kesavan J, Takashima Y, Ladewig J, Alexander M, et al. Capture of neuroepithelial-like stem cells from pluripotent stem cells provides a versatile system for in vitro production of human neurons. PloS one. 2012;7(1):e29597.

6. Chambers SM, Fasano CA, Papapetrou EP, Tomishima M, Sadelain M, Studer L. Highly efficient neural conversion of human ES and iPS cells by dual inhibition of SMAD signaling. Nature biotechnology. 2009;27(3):275-80.

7. Lam M, Moslem M, Bryois J, Pronk RJ, Uhlin E, Ellstrom ID, et al. Single cell analysis of autism patient with bi-allelic NRXN1-alpha deletion reveals skewed fate choice in neural progenitors and impaired neuronal functionality. Experimental cell research. 2019;383(1):111469.

8. Piovani G, Lanzi G, Ferraro RM, Masneri S, Barisani C, Savio G, et al. Generation of induced pluripotent stem cells (iPSCs) from patient with Cri du Chat Syndrome. Stem cell research. 2019;35:101393.

9. Shi Y, Kirwan P, Smith J, Robinson HP, Livesey FJ. Human cerebral cortex development from pluripotent stem cells to functional excitatory synapses. Nature neuroscience. 2012;15(3):477-86, S1.

10. Menendez L, Kulik MJ, Page AT, Park SS, Lauderdale JD, Cunningham ML, et al. Directed differentiation of human pluripotent cells to neural crest stem cells. Nature protocols. 2013;8(1):203-12.

11. Adamo A, Atashpaz S, Germain PL, Zanella M, D'Agostino G, Albertin V, et al. 7q11.23 dosage-dependent dysregulation in human pluripotent stem cells affects transcriptional programs in disease-relevant lineages. Nature genetics. 2015;47(2):132-41.

12. Frega M, Linda K, Keller JM, Gumus-Akay G, Mossink B, van Rhijn JR, et al. Neuronal network dysfunction in a model for Kleefstra syndrome mediated by enhanced NMDAR signaling. Nature communications. 2019;10(1):4928.

13. Willemsen MH, Beunders G, Callaghan M, de Leeuw N, Nillesen WM, Yntema HG, et al. Familial Kleefstra syndrome due to maternal somatic mosaicism for interstitial 9q34.3 microdeletions. Clinical genetics. 2011;80(1):31-8.

14. Ball MP, Li JB, Gao Y, Lee JH, LeProust EM, Park IH, et al. Targeted and genome-scale strategies reveal gene-body methylation signatures in human cells. Nature biotechnology. 2009;27(4):361-8.

15. Si-Tayeb K, Noto FK, Sepac A, Sedlic F, Bosnjak ZJ, Lough JW, et al. Generation of human induced pluripotent stem cells by simple transient transfection of plasmid DNA encoding reprogramming factors. BMC developmental biology. 2010;10:81.

16. Mariani J, Simonini MV, Palejev D, Tomasini L, Coppola G, Szekely AM, et al. Modeling human cortical development in vitro using induced pluripotent stem cells. Proceedings of the National Academy of Sciences of the United States of America. 2012;109(31):12770-5.

17. Abyzov A, Mariani J, Palejev D, Zhang Y, Haney MS, Tomasini L, et al. Somatic copy number mosaicism in human skin revealed by induced pluripotent stem cells. Nature. 2012;492(7429):438-42.

18. Mariani J, Coppola G, Zhang P, Abyzov A, Provini L, Tomasini L, et al. FOXG1-Dependent Dysregulation of GABA/Glutamate Neuron Differentiation in Autism Spectrum Disorders. Cell. 2015;162(2):375-90.

19. Kim JE, O'Sullivan ML, Sanchez CA, Hwang M, Israel MA, Brennand K, et al. Investigating synapse formation and function using human pluripotent stem cell-derived neurons. Proceedings of the National Academy of Sciences of the United States of America. 2011;108(7):3005-10.

20. Tai DJ, Ragavendran A, Manavalan P, Stortchevoi A, Seabra CM, Erdin S, et al. Engineering microdeletions and microduplications by targeting segmental duplications with CRISPR. Nature neuroscience. 2016;19(3):517-22.

21. Gillentine MA, Yin J, Bajic A, Zhang P, Cummock S, Kim JJ, et al. Functional Consequences of CHRNA7 Copy-Number Alterations in Induced Pluripotent Stem Cells and Neural Progenitor Cells. American journal of human genetics. 2017;101(6):874-87.

22. Deneault E, White SH, Rodrigues DC, Ross PJ, Faheem M, Zaslavsky K, et al. Complete Disruption of Autism-Susceptibility Genes by Gene Editing Predominantly Reduces Functional Connectivity of Isogenic Human Neurons. Stem cell reports. 2018;11(5):1211-25.

23. Zhang SC, Wernig M, Duncan ID, Brustle O, Thomson JA. In vitro differentiation of transplantable neural precursors from human embryonic stem cells. Nature biotechnology. 2001;19(12):1129-33.

24. Deshpande A, Yadav S, Dao DQ, Wu ZY, Hokanson KC, Cahill MK, et al. Cellular Phenotypes in Human iPSC-Derived Neurons from a Genetic Model of Autism Spectrum Disorder. Cell reports. 2017;21(10):2678-87.

25. Bilican B, Livesey MR, Haghi G, Qiu J, Burr K, Siller R, et al. Physiological normoxia and absence of EGF is required for the long-term propagation of anterior neural precursors from human pluripotent cells. PloS one. 2014;9(1):e85932.

26. Stacpoole SR, Bilican B, Webber DJ, Luzhynskaya A, He XL, Compston A, et al. Efficient derivation of NPCs, spinal motor neurons and midbrain dopaminergic neurons from hESCs at 3% oxygen. Nature protocols. 2011;6(8):1229-40.

27. Lancaster MA, Knoblich JA. Generation of cerebral organoids from human pluripotent stem cells. Nature protocols. 2014;9(10):2329-40.

28. Lancaster MA, Renner M, Martin CA, Wenzel D, Bicknell LS, Hurles ME, et al. Cerebral organoids model human brain development and microcephaly. Nature. 2013;501(7467):373-9.

29. Johnstone M, Vasistha NA, Barbu MC, Dando O, Burr K, Christopher E, et al. Reversal of proliferation deficits caused by chromosome 16p13.11 microduplication through targeting NFkappaB signaling: an integrated study of patient-derived neuronal precursor cells, cerebral organoids and in vivo brain imaging. Molecular psychiatry. 2019;24(2):294-311.

30. Pasca SP, Portmann T, Voineagu I, Yazawa M, Shcheglovitov A, Pasca AM, et al. Using iPSC-derived neurons to uncover cellular phenotypes associated with Timothy syndrome. Nature medicine. 2011;17(12):1657-62.

31. Yazawa M, Hsueh B, Jia X, Pasca AM, Bernstein JA, Hallmayer J, et al. Using induced pluripotent stem cells to investigate cardiac phenotypes in Timothy syndrome. Nature. 2011;471(7337):230-4.

32. Gaspard N, Bouschet T, Herpoel A, Naeije G, van den Ameele J, Vanderhaeghen P. Generation of cortical neurons from mouse embryonic stem cells. Nature protocols. 2009;4(10):1454-63.

33. Shcheglovitov A, Shcheglovitova O, Yazawa M, Portmann T, Shu R, Sebastiano V, et al. SHANK3 and IGF1 restore synaptic deficits in neurons from 22q13 deletion syndrome patients. Nature. 2013;503(7475):267-71.

34. Cocks G, Curran S, Gami P, Uwanogho D, Jeffries AR, Kathuria A, et al. The utility of patient specific induced pluripotent stem cells for the modelling of Autistic Spectrum Disorders. Psychopharmacology. 2014;231(6):1079-88.

35. Kathuria A, Nowosiad P, Jagasia R, Aigner S, Taylor RD, Andreae LC, et al. Stem cell-derived neurons from autistic individuals with SHANK3 mutation show morphogenetic abnormalities during early development. Molecular psychiatry. 2018;23(3):735-46.

36. Hotta A, Cheung AY, Farra N, Garcha K, Chang WY, Pasceri P, et al. EOS lentiviral vector selection system for human induced pluripotent stem cells. Nature protocols. 2009;4(12):1828-44.

37. Brennand KJ, Simone A, Jou J, Gelboin-Burkhart C, Tran N, Sangar S, et al. Modelling schizophrenia using human induced pluripotent stem cells. Nature. 2011;473(7346):221-5.

38. Djuric U, Cheung AYL, Zhang W, Mok RS, Lai W, Piekna A, et al. MECP2e1 isoform mutation affects the form and function of neurons derived from Rett syndrome patient iPS cells. Neurobiology of disease. 2015;76:37-45.

39. Ross PJ, Zhang WB, Mok RSF, Zaslavsky K, Deneault E, D'Abate L, et al. Synaptic Dysfunction in Human Neurons With Autism-Associated Deletions in PTCHD1-AS. Biological psychiatry. 2019.

40. Marchetto MC, Carromeu C, Acab A, Yu D, Yeo GW, Mu Y, et al. A model for neural development and treatment of Rett syndrome using human induced pluripotent stem cells. Cell. 2010;143(4):527-39.

41. Machado CO, Griesi-Oliveira K, Rosenberg C, Kok F, Martins S, Passos-Bueno MR, et al. Collybistin binds and inhibits mTORC1 signaling: a potential novel mechanism contributing to intellectual disability and autism. European journal of human genetics : EJHG. 2016;24(1):59-65.

42. Espuny-Camacho I, Michelsen KA, Gall D, Linaro D, Hasche A, Bonnefont J, et al. Pyramidal neurons derived from human pluripotent stem cells integrate efficiently into mouse brain circuits in vivo. Neuron. 2013;77(3):440-56.

43. Nageshappa S, Carromeu C, Trujillo CA, Mesci P, Espuny-Camacho I, Pasciuto E, et al. Altered neuronal network and rescue in a human MECP2 duplication model. Molecular psychiatry. 2016;21(2):178-88.
