# Supplementary material for: Copy number variants (CNVs): a powerful tool for iPSC-based modelling of ASD
Source: Mol Autism. 2020 Jun 1;11:42. doi: 10.1186/s13229-020-00343-4 (PMC7268297; doi:10.1186/s13229-020-00343-4)
Supplement: Supplementary file 2 — Additional file 2: Supplementary Table 2b. iPSCs generated from patients not diagnosed with ASD but carrying CNVs which might be associated with NDD. [file 13229_2020_343_MOESM2_ESM.docx]

**Supplementary Table 2b. iPSCs generated from patients not diagnosed with ASD but carrying CNVs which might be associated with NDD**

| **CNV** | **Type and size of CNV** | **Source** | **Reprogramming** | **Number of patients and healthy controls** | **Main clinical features of the patients** | **Differentiation protocol** | **Neuronal cell types** | **Time from taken the somatic cells to phenotype neurons** | **Associated cellular phenotype** | **Validation and quality control** | **Isogenic**  **cell lines** | **Ref** |
| --- | --- | --- | --- | --- | --- | --- | --- | --- | --- | --- | --- | --- |
| 3p26.3 | ~ 1 Mb microduplication (begins approximately 600 kb upstream of the *CNTN6* gene and ends more than 50 kb downstream of  its stop codon) | fibroblasts | LeGO lentiviral vectors containing *OCT4*,  *SOX2*, C-*MYC* and *KLF4* | patient: 1 (number of clones 7)  controls: 2 (number of clones at least 7) | intellectual disability | neurons (Ngn2 overexpression protocol ([1](#_ENREF_1)))  neurons (through neural rosette stage ([2](#_ENREF_2))) | Neural progenitors  layer 2/3 excitatory  cortical neurons | from fibroblasts to iPSCs - 18 days  from iPSCs to neurons - 3 weeks (Ngn2 overexpression protocol)  from iPSC to neurons - ~ 40 days (through neural rosette stage) | neurons from patient and control had similar characteristics with respect to neuronal markers and electrophysiological activities  reduced expression of *CNTN6* gene in patient neurons compared to  control neurons | morphology  karyotype analysis  expression of pluripotency markers (OCT4, NANOG, TRA-1-60, SSEA4)  teratoma assay  microsatellite  analysis | No | ([3](#_ENREF_3)) |
| 15q11.2 | ∼ 382 kb microdeletion between BP1 and BP2 | fibroblasts | Sendai virus (five constructs TS7-*OCT3/4*, -*SOX2*, -*KLF4*, -c-*MYC*, -GFP) | patients: 2  control: 1 | patient 1 - schizoaffective disorder  patient 2 (mother of patient no. 1) - / | neurons ([4](#_ENREF_4)) | neural progenitor cells  neurons | from fibroblasts to iPSCs - not available in the paper  from iPSCs to neural rosettes - ~ 1 week  from NPCs to neurons - 10 weeks | dendritic morphology was altered in patient neurons compared to control cells | aCGH | No | ([5](#_ENREF_5)) |
| 15q11.2- q13.1 | ~5.57 Mb  (chr15:21,144,837-  26,722,409) duplication | peripheral blood mononuclear cells | episomal vectors (pCE-hOCT3/4, pCE-hSK, pCE-hUL, pCE-mp53DD, and pCXB-EBNA1) | patient: 1 (3 iPSC lines) | delay in psychomotor development  severe intellectual disability  multiple  social and interpersonal problems | / | / | from peripheral blood mononuclear cells to iPSCs – 26 days | / | Morphology  analysis of pluripotency markers (NANOG and TRA-1-60) expression  capacity to differentiate into three germ layers *in vitro*  aCGH  STR analysis  mycoplasma testing | No | ([6](#_ENREF_6)) |
| 15q11- q13.1 | isodicentric and interstitial duplications of  15q11-q13 | fibroblasts  umbilical cord blood | - retroviral, lentiviral or episomal vectors encoding *OCT4*, *SOX2*, *KLF4*, *MYC* and *LIN28* | patients: 4 (two individual with idic (isodicentric), one with a paternally-inherited duplication of chromosome 15q11-q13.1, and one who was mosaic for maternally-inherited interstitial duplication of chromosome 15q11-q13.1) (5 iPSCs cell lines)  control: 1 | / | neuron (embryoid body-based protocol or monolayer differentiation ([7](#_ENREF_7), [8](#_ENREF_8)) | vesicular glutamate transporter  1 -positive excitatory neurons  glutamate  decarboxylate 65 -positive inhibitory  neurons | from iPSCs to neural progenitors – 3 weeks  from iPSCs to neurons - 10 weeks | downregulation of genes involved in neuron  development in patient neurons compared to normal neurons  genes involved in cell cycle and protein catabolic processes were upregulated in idic(15) neurons | morphology  karyotype analysis  expression of pluripotency markers (OCT4, SOX2, LIN28, NANOG, SSEA4, TRA1-60, FGF4)  analysis of multipotency  analysis of methylation at Prader-Willi syndrome imprinting center  allele-specific single nucleotide polymorphism analysis  FISH with a probe for the SNRPN gene and a control probe in the distal long arm of chromosome 15  whole genome copy number analysis using the Affymetrix  CytoScan HD Array  array CGH | No | ([9](#_ENREF_9)) |
| 15q13.3 | heterozygous  15q13.3 deletions and duplications  Patient 1 - BP3/BP5 duplication  Patient 2 - D-CHRNA7-LCR/BP5 duplication  Patient 3 - BP4/BP5 deletion | fibroblasts | CytoTune-iPS Sendai Reprogramming Kit | patients: 3  controls: 3  (2-3 iPSC clones per individual) | patient 1 - intellectual disability/developmental delay (ID/DD); attention  deficit-hyperactivity disorder  patient 2 - /  patient 3 – ID/DD | neural progenitor cells (dual SMAD inhibition protocol ([10](#_ENREF_10), [11](#_ENREF_11))) | cortical-like neural progenitor cells | from fibroblast to iPSCs colonies- ~ 21 days  from iPSCs to NPCs - 12 days | α7 nicotinic acetylcholine receptor (α7 nAChR)- associated calcium flux was decreased in 15q13.3 deletion and duplication probands  gene expression of chaperones involved in folding, assembly and trafficking α7 nAChRs was increased in 15q13.3 duplication NPCs  expression of a subset of ER stress markers was increased in 15q13.3 duplication NPCs  decreased expression of JAK2 is observed in both CNV groups | karyotype analysis  expression of pluripotency markers (OCT4, SSEA-4, SOX2) | Tai et al. generated 15q13.3 microduplication and microdeletion (2 Mb) via CRISPR/Cas9 ([12](#_ENREF_12)) | ([13](#_ENREF_13)) |
| 17p13.3 | Patient 1 – 4.5 Mb deletion  Patient 2 – 5.7 Mb deletion  Patient 3 – 2.7 Mb deletion | fibroblasts | episomal - plasmids encoding *OCT3/4*, *SOX2*, *KLF4*, *L-MYC*, *LIN28* and shRNA for *TP53* | patients: 3 (2 clones each)  controls: 3 (1 or 2 clones per control) | Miller Dieker Syndrome | Cerebral organoids ([14](#_ENREF_14)) | Cerebral organoids (neuroepithelial stem cells, neurons) | from somatic cells to iPSCs - ~ 25 days  from iPSCs to organoids - 2.5 - 10 weeks | patient organoids have reduced size  increased apoptosis in the patient cortical VZ-like regions  decreased vertical divisions in patient  organoids  defective neuronal migration in patient organoids  increased abundance of deep-layer neurons in patient organoids  mitotic defect (delay in cell division) in  patient outer radial glia cells | morphology  karyotyping  analysis of episomal transgene expression  teratoma formation assays  differentiation potential  expression of pluripotency markers | No | ([15](#_ENREF_15)) |
| Xq28 | Patient 1 - 300 kb duplication (Xq28 (152.73–153.02 Mb))  Patient 2 - 15.25 Mb (Xq28 (139.33–154.58 Mb)) duplication | fibroblasts | pMXs retroviral vectors containing *OCT4*, *SOX2*, *KLF4* and *C-MYC* | patients: 2  controls: 2 healthy persons and BJ1 fibroblasts | patient 1 - severely delayed development; no active speech; repetitive behavior; epilepsy  patient 2 - non-verbal with severe stereotypic behaviour | neurons ([16](#_ENREF_16)) | forebrain progenitors  pyramidal neurons | from cultivated fibroblast to iPSCs –3-4 weeks  from iPSCs to neurons – 30-60 days | increase synaptogenesis and dendritic complexity in patient cortical neurons  altered neuronal network synchronization in patient-derived neurons | expression of pluripotent genes  analysis of potency to generate three germ layers  teratoma formation assay  array CGH  karyotype analysis | No | ([17](#_ENREF_17)) |

**References**

1. Zhang Y, Pak C, Han Y, Ahlenius H, Zhang Z, Chanda S, et al. Rapid single-step induction of functional neurons from human pluripotent stem cells. Neuron. 2013;78(5):785-98.

2. Muratore CR, Srikanth P, Callahan DG, Young-Pearse TL. Comparison and optimization of hiPSC forebrain cortical differentiation protocols. PloS one. 2014;9(8):e105807.

3. Gridina MM, Matveeva NM, Fishman VS, Menzorov AG, Kizilova HA, Beregovoy NA, et al. Allele-Specific Biased Expression of the CNTN6 Gene in iPS Cell-Derived Neurons from a Patient with Intellectual Disability and 3p26.3 Microduplication Involving the CNTN6 Gene. Molecular neurobiology. 2018;55(8):6533-46.

4. D'Aiuto L, Prasad KM, Upton CH, Viggiano L, Milosevic J, Raimondi G, et al. Persistent infection by HSV-1 is associated with changes in functional architecture of iPSC-derived neurons and brain activation patterns underlying working memory performance. Schizophrenia bulletin. 2015;41(1):123-32.

5. Das DK, Tapias V, D'Aiuto L, Chowdari KV, Francis L, Zhi Y, et al. Genetic and morphological features of human iPSC-derived neurons with chromosome 15q11.2 (BP1-BP2) deletions. Molecular neuropsychiatry. 2015;1(2):116-23.

6. Arioka Y, Kushima I, Mori D, Ozaki N. Three lines of induced pluripotent stem cells derived from a 15q11.2-q13.1 duplication syndrome patient. Stem cell research. 2018;31:240-3.

7. Germain ND, Banda EC, Becker S, Naegele JR, Grabel LB. Derivation and isolation of NKX2.1-positive basal forebrain progenitors from human embryonic stem cells. Stem cells and development. 2013;22(10):1477-89.

8. Pankratz MT, Li XJ, Lavaute TM, Lyons EA, Chen X, Zhang SC. Directed neural differentiation of human embryonic stem cells via an obligated primitive anterior stage. Stem cells. 2007;25(6):1511-20.

9. Germain ND, Chen PF, Plocik AM, Glatt-Deeley H, Brown J, Fink JJ, et al. Gene expression analysis of human induced pluripotent stem cell-derived neurons carrying copy number variants of chromosome 15q11-q13.1. Molecular autism. 2014;5:44.

10. Chambers SM, Fasano CA, Papapetrou EP, Tomishima M, Sadelain M, Studer L. Highly efficient neural conversion of human ES and iPS cells by dual inhibition of SMAD signaling. Nature biotechnology. 2009;27(3):275-80.

11. Kim JE, O'Sullivan ML, Sanchez CA, Hwang M, Israel MA, Brennand K, et al. Investigating synapse formation and function using human pluripotent stem cell-derived neurons. Proceedings of the National Academy of Sciences of the United States of America. 2011;108(7):3005-10.

12. Tai DJ, Ragavendran A, Manavalan P, Stortchevoi A, Seabra CM, Erdin S, et al. Engineering microdeletions and microduplications by targeting segmental duplications with CRISPR. Nature neuroscience. 2016;19(3):517-22.

13. Gillentine MA, Yin J, Bajic A, Zhang P, Cummock S, Kim JJ, et al. Functional Consequences of CHRNA7 Copy-Number Alterations in Induced Pluripotent Stem Cells and Neural Progenitor Cells. American journal of human genetics. 2017;101(6):874-87.

14. Kadoshima T, Sakaguchi H, Nakano T, Soen M, Ando S, Eiraku M, et al. Self-organization of axial polarity, inside-out layer pattern, and species-specific progenitor dynamics in human ES cell-derived neocortex. Proceedings of the National Academy of Sciences of the United States of America. 2013;110(50):20284-9.

15. Bershteyn M, Nowakowski TJ, Pollen AA, Di Lullo E, Nene A, Wynshaw-Boris A, et al. Human iPSC-Derived Cerebral Organoids Model Cellular Features of Lissencephaly and Reveal Prolonged Mitosis of Outer Radial Glia. Cell stem cell. 2017;20(4):435-49 e4.

16. Espuny-Camacho I, Michelsen KA, Gall D, Linaro D, Hasche A, Bonnefont J, et al. Pyramidal neurons derived from human pluripotent stem cells integrate efficiently into mouse brain circuits in vivo. Neuron. 2013;77(3):440-56.

17. Nageshappa S, Carromeu C, Trujillo CA, Mesci P, Espuny-Camacho I, Pasciuto E, et al. Altered neuronal network and rescue in a human MECP2 duplication model. Molecular psychiatry. 2016;21(2):178-88.
